# Long-Distance Retinoid Signaling in the Zebra Finch Brain

**DOI:** 10.1371/journal.pone.0111722

**Published:** 2014-11-13

**Authors:** Tina C. Roeske, Constance Scharff, Christopher R. Olson, Arpik Nshdejan, Claudio V. Mello

**Affiliations:** 1 Department of Psychology, Hunter College, City University of New York, New York, New York, United States of America; 2 Department of Animal Behavior, Freie Universität Berlin, Berlin, Germany; 3 Department of Behavioral Neuroscience, Oregon Health and Science University, Portland, Oregon, United States of America; Laboratoire de Biologie du Développement de Villefranche-sur-Mer, France

## Abstract

All-trans retinoic acid (ATRA), the main active metabolite of vitamin A, is a powerful signaling molecule that regulates large-scale morphogenetic processes during vertebrate embryonic development, but is also involved post-natally in regulating neural plasticity and cognition. In songbirds, it plays an important role in the maturation of learned song. The distribution of the ATRA-synthesizing enzyme, zRalDH, and of ATRA receptors (RARs) have been described, but information on the distribution of other components of the retinoid signaling pathway is still lacking. To address this gap, we have determined the expression patterns of two obligatory RAR co-receptors, the retinoid X receptors (RXR) α and γ, and of the three ATRA-degrading cytochromes CYP26A1, CYP26B1, and CYP26C1. We have also studied the distribution of zRalDH protein using immunohistochemistry, and generated a refined map of ATRA localization, using a modified reporter cell assay to examine entire brain sections. Our results show that (1) ATRA is more broadly distributed in the brain than previously predicted by the spatially restricted distribution of zRalDH transcripts. This could be due to long-range transport of zRalDH enzyme between different nuclei of the song system: Experimental lesions of putative zRalDH peptide source regions diminish ATRA-induced transcription in target regions. (2) Four telencephalic song nuclei express different and specific subsets of retinoid-related receptors and could be targets of retinoid regulation; in the case of the lateral magnocellular nucleus of the anterior nidopallium (lMAN), receptor expression is dynamically regulated in a circadian and age-dependent manner. (3) High-order auditory areas exhibit a complex distribution of transcripts representing ATRA synthesizing and degrading enzymes and could also be a target of retinoid signaling. Together, our survey across multiple connected song nuclei and auditory brain regions underscores the prominent role of retinoid signaling in modulating the circuitry that underlies the acquisition and production of learned vocalizations.

## Introduction

All-trans retinoic acid (ATRA) acts as a transcriptional regulator in many tissues. It is best known for its role as a morphogen in vertebrate embryonic development [1], but an increasing body of evidence shows that ATRA remains active in the postnatal brain [2], where it is involved in numerous plasticity-related processes. ATRA is implicated in long-term potentiation [3]–[5], other forms of synaptic plasticity and homeostasis, including the regulation of synaptic AMPA and GABA_A_ receptor trafficking [6], neurogenesis [7]–[11], spatial learning and memory [3], [12]–[17], and modulation of age-related cognitive decline [15], [18], [19].

**Figure 1:**
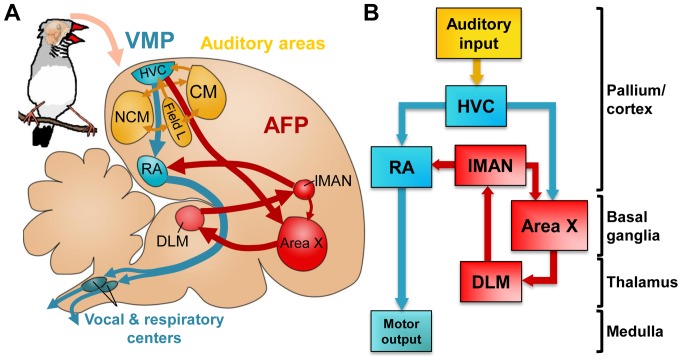
Neural substrates for singing and song learning. A and B: schematics of the song control system and relevant auditory structures. Represented are the posterior vocal-motor pathway (VMP, blue), and the anterior forebrain pathway (AFP, red). HVC is the origin of both pathways and the entry site of inputs from auditory areas (yellow) into the song system. For abbreviations, see table 1. In A, brain topography is preserved, for easier comparison with experimental brain sections. In B, indication of broad brain subdivisions (on the right) facilitates comparison with mammalian brains.

The songbird is a particularly interesting model to examine the role that retinoids play in postnatal behavioral plasticity [20], because song is a complex learned vocal behavior that depends on retinoid signaling for its normal development [21]. Furthermore, the set of discrete brain nuclei that subserve the acquisition and production of song (a.k.a. ‘the song system’) is well characterized anatomically and functionally. This system has two main subdivisions (fig. 1; reviewed by Prather [22]): 1) the posterior vocal-motor pathway (VMP), comprising the nidopallial nucleus HVC (used as proper name; for abbreviations, see table 1), the robust nucleus of the arcopallium (RA), and brainstem vocal and respiratory centers; and 2) the anterior forebrain pathway (AFP), consisting of a pallial—basal-ganglia—thalamo—pallial loop that includes striatal Area X, the medial part of the dorsolateral thalamic nucleus (DLM) and the lateral magnocellular nucleus of the anterior nidopallium (lMAN). The two pathways are connected through HVC-to-Area X and LMAN-to-RA projections. The vocal-motor pathway is essential for singing, whereas the AFP is required for song acquisition in juvenile birds and for modulating song variability and auditory-dependent plasticity in both juvenile and adult birds, as reviewed by Gale and Perkel [23]. HVC is an important node in the song system since it gives rise to both the posterior and anterior pathways, and it is also a major entry site for auditory information [22]. High-order auditory areas like the caudomedial nidopallium (NCM) and the caudal mesopallium (CM) receive input from the primary auditory telencephalic area, field L, and are involved in the perceptual processing and/or memorization of birdsong [22].

**Table 1:** Abbreviations for zebra finch brain structures.

A	Arcopallium	lMAN	lateral magnocellular nucleus of anterior nidopallium
Area X	Area X of the medial striatum	MLd	dorsal part of nucleus mesencephalicus lateralis
Cb	cerebellum	N	Nidopallium
CLM	caudolateral mesopallium	NC	caudal nidopallium
CM	caudal mesopallium	NCM	caudomedial nidopallium
CMM	caudomedial mesopallium	NIII	oculomotor nerve
DLM	dorsolateral medial nucleus of the thalamus	M	Mesopallium
DMP	nucleus dorsomedialis posterior of the thalamus	Ov	nucleus ovoidalis
E	entopallium	pHVC	para-HVC
GP	globus pallidus	PMI	nucleus paramedianus internus of the thalamus
H	hyperpallium	RA	robust nucleus of the arcopallium
Hbm	nucleus habenularis medialis	S	Septum
HA	hyperpallium apicale	SNC	compact part of substantia nigra
HD	hyperpallium densocellulare	SpL	nucleus spiriformis lateralis
Hp	hippocampus	SpM	nucleus spiriformis medialis
HVC	nucleus HVC of the nidopallium	St	striatum
ICo	nucleus intercollicularis	TeO	optic tectum
IM	magnocellular part of nucleus isthmi	TrO	tractus opticus
IPC	parvocellular part of nucleus isthmi	V	ventricle
LAD	lamina of the dorsal arcopallium	VTA	ventral tegmental area
L2a	subfield L2a of field L		

**Figure 2:**
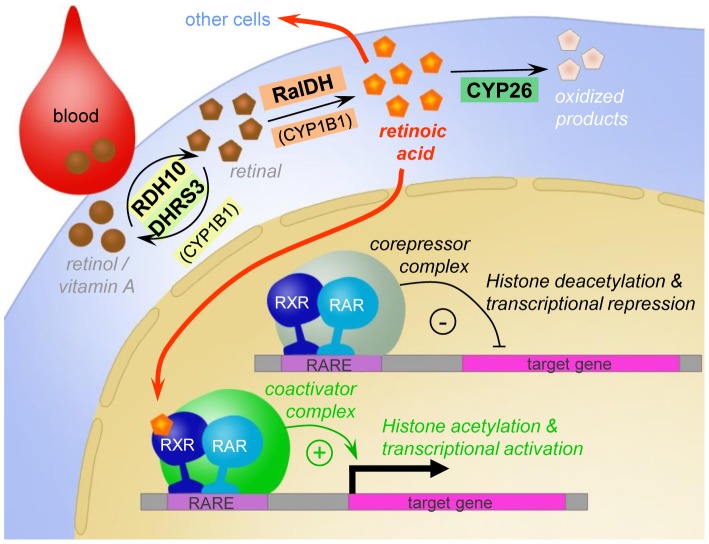
Simplified schematic of the all-trans retinoic acid (ATRA) signaling pathway. ATRA is synthesized from retinol (Vitamin A) in a two-step oxidation process which takes place in the cytoplasm. To induce transcription, retinoic acid receptors (RARs) need to be activated by ATRA binding, and both RARs and retinoid X receptors (RXRs) need to bind retinoic acid response elements (RAREs) in the vicinity of target genes. Several CYP26 isoforms are responsible for ATRA degradation. Due to its small size and hydro- and lipophilic properties, ATRA is able to cross cell membranes and thus act in other cells.

The targets of ATRA in the brain are dependent on the distribution of its synthetic enzymes, target receptors and degradation enzymes which work in concert to locally control levels of ATRA, and knowing how these systems operate in the songbird brain would provide us an inference into how retinoid signaling may regulate the ability of a bird to learn its song. In the postnatal zebra finch brain ATRA activity has been associated with the expression of retinaldehyde-specific aldehyde dehydrogenase (zRalDH, a.k.a. RalDH2, or Aldh1a2 [21], see fig. 2). Yet, other enzymes can oxidize retinal to retinoic acid: aldehyde dehydrogenases RalDH1, RalDH3 and, as recently shown, a cytochrome, CYP1B1, can also mediate this reaction (see [1] for a review). Thus, while zRalDH is likely a good indicator of ATRA synthesis in the post-natal songbird brain, we cannot exclude the possible activity of other ATRA synthesizing enzymes whose brain expression patterns remain unknown. However, for HVC, a combination of immunodetection and enzyme kinetics methods has not revealed any evidence for the presence of multiple related ATRA synthesizing enzyme types [24], suggesting that zRalDH likely constitutes the major enzyme involved in ATRA synthesis in major song nuclei of the zebra finch brain. zRalDH mRNA in adult zebra finches is expressed in a very restricted pattern, primarily in HVC and in a rostral nidopallial band that includes lMAN [21], [25], [26]. This suggests that ATRA is produced at discrete brain sites, a possibility supported by evidence from cell reporter assays using tissue punches [21]. Local pharmacological blocking of zRalDH activity in the HVC of juvenile males disrupts normal song maturation, indicating a critical role for the ATRA synthesized in this song nucleus [21]. However, the exact sites of retinoid action relevant for song development are not known. Due to its hydro-lipophilic properties, ATRA can potentially reach brain areas several hundred micrometers away from its production sites [27], [28]. Consistent with this possibility, the brain distribution of the retinoic acid receptors (RARs) α, β, and γ, as determined by *in situ* hybridization (ISH), is very broad [29], and includes areas that are distant from sites of zRalDH expression. Importantly, RARs need to form heterodimers with another class of nuclear receptors, retinoid X receptors (RXRs), before they can act as transcriptional regulators upon binding of ATRA [30]–[34], as depicted in fig. 2. Thus, determining the distribution of RXRs is essential to identify actual target sites of ATRA signaling. Furthermore, the cytochromes CYP26A1, CYP26B1, and CYP26C1 are the main enzymes responsible for ATRA degradation (fig. 2) [35]–[37], thus their brain distribution limits the spatial extent of ATRA action.

Because the distribution of RXRs and the retinoic acid-degrading cytochromes in the songbird brain is unknown, we cloned the zebra finch orthologs of RXRs and of the known retinoic-acid degrading cytochromes, and mapped their distribution by ISH in juvenile and adult zebra finch brains. We also generated a more refined brain distribution map for ATRA localization, by use of a modified cell culture reporter assay to visualize ATRA-induced LacZ expression on entire brain sections. Our results implicate song nuclei RA and Area X as major targets for ATRA signaling, although these areas are distant from zRalDH transcription sites. In CM, the retinoic acid degradation enzyme CYP26B1 was expressed in a conspicuous gradient-like pattern, indicating that ATRA levels may also be under tight regulation in a major auditory forebrain area. Overall, our results together with previous findings generate a comprehensive picture of ATRA signaling components in the songbird brain.

## Materials and Methods

### Animals

Male zebra finches (*Taeniopygia guttata*) were obtained from breeding colonies at the Freie Universität Berlin and the Max Planck Institute for Molecular Genetics, Berlin. Birds were housed in family or group cages in a breeding room with a 12 12h light-dark cycle. We used a total of 68 zebra finches (50 adult males, >120 days old; 2 male juveniles, 20 days old; 5 male juveniles, 38–49 days old; 2 male juveniles, 50–51 days old; 4 male juveniles, 64–68 days old; 4 adult females, >120 days old; 1 embryo).

Tables S1, S2, and S3 indicate the number of animals per experiment, their age, and treatments.

### Ethics Statement

Experiments were in accordance to Institutional guidelines and approved by the German equivalent to the IACUC (*Landesamt für Arbeitsschutz, Gesundheit und technische Sicherheit Berlin*).

### In situ hybridization probes for the RXRs and retinoic acid-degrading cytochromes (CYP26s)

Brains of a total of 34 male and 2 female zebra finches, as well as one whole embryo, were subjected to ISH (see table S1 for details).

#### Generating probes

A cDNA from the ESTIMA database (Songbird Neurogenomics Initiative; http://titan.biotec.uiuc.edu/songbird) was available for RXRα and was used for expression analysis. For all other genes examined, no clones were available in ESTIMA. We therefore PCR-cloned fragments corresponding to their zebra finch orthologs. For this purpose, we first isolated total brain RNA from an adult male zebra finch using the TRIzol method. We then used reverse transcriptase SuperScript II (Invitrogen) to transcribe total brain RNA, synthesized second strands using T4 DNA polymerase, digested RNA with RNAse H, and used the first-strand cDNA directly as a PCR template with gene specific primers. We designed primer pairs for RXRγ and CYP26A1 based on the genomic sequence, aided by examination of conserved regions identified through the alignment of the orthologs from several species deposited in GenBank, if genomic sequence quality was poor. The primer sequences were as follows: For RXRγ, forward 5′-GGGAAGCACTATGGGGTGTA-3′, reverse 5′-CTGATCGACAAGCGCCAGCG-3′, predicted fragment length 799 bp. For CYP26A1: forward 5′-CTGAATGAGTCTGCCACAG-3′, reverse 5′-CTTCATGTCTCCATCTCCAG-3′, predicted fragment length 406 bp. For CYP26B1 and CYP26C1, primer sequences for cloning were based on primer sequences previously used for chicken CYP26B1 and CYP26C1 [38], [39]. All PCR products were examined on agarose gels, purified with the Qiaquick PCR purification kit (Qiagen) and inserted into pGEM-T easy vectors (Promega); resulting clones were sequenced for identification, and full-length ORF sequences were deposited in GenBank under the following accession numbers: RXRα: HQ830555; RXRγ: HQ830557; Cyp26A1: HQ830558; Cyp26B1: HQ830556; Cyp26C1: HQ830559 (for Cyp26C1, only a partial ORF sequence was cloned).

#### RXRα

ESTIMA zebra finch clone SB03015A2E11.f1 (GenBank accession #: DV949832) corresponds to the 3′ portion of the RXRα mRNA, extending from the middle of the coding region for the ligand-binding domain to the polyA tail (fig. S1.A). To assess the likelihood of cross-hybridization between this RXRα clone and other RXRs, we blat aligned the RXRα clone sequence against the entire zebra finch genome (UCSC browser). Whereas the EST sequence (514bp) fully aligns with the RXRα locus with 99.7% identity (blat score 1358), it has only a very short partial alignment (88bp) at the RXRγ locus at 89.7% similarity (blat score 71). Probe specificity for RXRα as opposed to RXRγ was further indicated by a total lack of overlap between the two expression patterns.

#### RXRγ

The amplified fragment was 738 bp long and spanned large parts of the zinc finger and the ligand-binding domains (fig. S1.B). It corresponds to the shorter of two transcript variants that have been described in chicken; it also corresponds to the only known RXRγ variant in mammals, such as human, mouse, and rat (UCSC genome browser). This probe produced distinct and sparse expression patterns on both brain and embryonic sections. As in chicken and Xenopus embryos [40]–[42], RXRγ expression in zebra finch embryos was highest in the eye cup (data not shown), providing further evidence of probe specificity.

#### CYP26A1

The fragments used as templates for *in-situ* hybridization were 590 and 406 bp long and corresponded to 5′ and 3′ domains respectively (fig. S2.A). Blasting these sequences against the zebra finch genome resulted in highest scores of less than 50%, and those were for the other zebra finch CYP26 family members. Thus, cross-hybridization of the probes is highly unlikely.

#### CYP26B1 and CYP26C1

We generated CYP26B1 and CYP26C1 riboprobes by cloning fragments using primer sequences provided by Reijntjes et al. based on chicken [39], [43]. We obtained a 386 bp fragment for CYP26B1 that maps close to the 5′ end of the gene, and a 495 bp fragment for CYP26C1, that maps to the middle of the open reading frame (fig. S2.B,C).

### Labeling of riboprobes

Plasmids containing fragments of interest were isolated from bacteria using Qiagen's miniprep kit (Qiagen). Templates for *in vitro* transcription were re-amplified PCR products of the fragments of interest, using M13 primers. The cloning vector of the RXRα ESTIMA clone, pSport1 (GenBank Accession No. U12390), contained promoters for RNA polymerases T3 and T7; the cloning vector pGEM-T easy, which was used for all other fragments, contained promoters for SP6 and T7. The templates were purified using Qiagen's PCR purification kit, and sense and antisense ^33^P or digoxigenin-labeled riboprobes were generated using SP6, T3, or T7 RNA polymerases (Promega). For radioactive ^33^P-labeled riboprobes, the transcription buffer contained 50 mM DTT (Roche), 200mM Tris-HCl (Roth), 30mM MgCl_2_ (Roth), 50mM NaCl (Roth), and 10 mM spermidine (Roche). For transcription, we added template PCR product to a final concentration of 40 ng/µl; rATP, rCTP, and rGTP (Roche) to a final concentration of 0.5 mM each; 12µM rUTP (Roche), 2µCi/µl ^33^P-UTP (Amersham), 1µg/µl BSA (bovine serum albumin; Sigma), 5% (v/v) RNAse inhibitor (Amersham), and 10% (v/v) of the respective polymerase. For digoxigenin-labeled riboprobes, *in vitro* transcription was done with using a commercial buffer, adding 0.4 mM of each rNTP (Roche); 36% of the total UTP was digoxigenin labeled digUTP (Roche). 10mM DTT (Roche), and RNAse inhibitor and polymerase were added, as for radioactive *in vitro* transcription. Transcription reactions were run for 2h at 37°C, except for SP6 reactions, which were run at 40°C. Probes were purified using ProbeQuant G-50 Micro Columns (GE Healthcare) according to the manufacturer's directions.

### Radioactive and non-radioactive in situ hybridization

Adult and juvenile male zebra finches were overdosed with isoflurane, decapitated and their brains were quickly dissected and frozen over liquid nitrogen. Brains were cut at 14 or 16µm on a cryostat (Leica) and stored at −75°C. For ^33^P ISH, we followed a previously described protocol [44], [45], with slight modifications. Briefly, after fixation and dehydration through an alcohol series, the slides were acetylated for 10min at RT in 0.0135% triethanolamine (Merck) and 0.0025% acetic anhydride (Fluka) in water. After dehydration through another alcohol series, sections were hybridized overnight in hybridization buffer (50% formamide (Fluka), 1µl/µl BSA (Sigma), 1µl/µl Poly A (Sigma), 2µg/µl tRNA (Sigma) in 2X SSPE) containing sense or antisense riboprobes (5×10^5^ cpm per section). Hybridization temperatures were 65°C for the RXRα ESTIMA probe, 56°C for the CYP26B1 probe, and 53°C for RXRγ, CYP26A1, and CYP26C1 probes. Coverslips were removed by dipping slides in 2X SSPE; slides were then washed sequentially in 2X SSPE buffer (1h at RT) and 2X SSPE/50% formamide (Fluka; 1h at hybridization temperature), followed by two high-stringency washes in 0.1X SSPE (30min at hybridization temperature). For the RXRγ, CYP26A1, and CYP26C1 probes, these washes were followed by RNAse A treatment (for other probes, our high stringency ISH protocol yielded low enough background in the sense-strand hybridized controls so that RNAse A treatment, which degrades Nissl substance of neuronal cells, was unnecessary): incubation in TNE (Tris-NaCl-EDTA) buffer for 10min, incubation in TNE containing 20µg/ml RNAse A – Roche - for 30min, final wash for 10min in TNE, followed by dehydration in an alcohol series. Signal was detected by exposure to a phosphorimager screen (GE Healthcare) for six to ten days. Radiographic signal was measured by a Storm PhosphorImager (Molecular Dynamics), and ImageQuant analysis software (Molecular Dynamics) was used to analyze the images.

Digoxigenin ISH required the following modifications: It included an additional 1 h prehybridization step at 65°C in 2% (w/v) SDS (Roth), 2% (v/v) Blocking Reagent (Roche), 250µg/ml tRNA (Gibco), 100µg/ml heparin (Polyscience Europe), and 50% formamide (Fluka) in 5X SSC pH 4.5). Hybridization solution with 1–5% (v/v) of probe was heated to 85°C for 5min before application to the slides. After overnight hybridization, slides were rinsed in 5X SSC, followed by four washes at hybridization temperature (1X SSC/50% formamide for 30min, 2X SSC for 20min, 0.2X SSC for two times 20min). Digoxigenin labeling was detected immunohistochemically using Anti-DIG-AP (1 2500, Roche). For staining, we used standard NBT/BCIP staining (NBT  =  Nitro-Blue Tetrazolium Chloride; BCIP  = 5-Bromo-4-Chloro-3'-Indolyphosphate p-Toluidine Salt). Briefly, sections were prepared by incubation in NTMT buffer (pH 9.5) for 10min and then incubated in the dark in NBT/BCIP standard staining solution for several days (duration depending on probe and staining intensity as inspected by eye). Fresh staining solution was applied every day. Slides were then rinsed in NTMT, washed twice in Phosphate Buffered Saline (PBS), and coverslipped. For comparison of expression patterns, additional selected sections were hybridized with ^33^P and digoxigenin-labeled riboprobes for the ATRA-synthesizing enzyme zRalDH [21]; the probe was the same as in Denisenko-Nehrbass et al. [21] and required hybridization at 60°C.

### Analysis of RXRα expression in song nuclei to assess circadian and age effects on expression strength

#### Experimental conditions, and statistics

(i) To determine whether RXRα expression in song nuclei undergoes circadian variations, we sacrificed 19 male birds (aged 64–783 days after hatching (PHD), average age 455) within 20min after lights were on and before any singing took place (“morning condition”; N = 3, PHD 447,445,445), within ∼1–1.5 h before lights off (“evening condition”; N = 2, PHD 395 and 396), or between 2h after lights on and 2 h before lights off (“daytime” condition; N = 14, PHD 64–783, average 465.5). (ii) To assess the effect of the birds' age on RXRα expression in the telencephalic song nuclei we used multivariate analysis of covariance (MANCOVA) using Matlab (release 2012b, The MathWorks, Inc., Natick, Massachusetts). We used age as the covariate, and time of the day as qualitative variable.

#### Quantification of expression

To compare RXRα expression in song nuclei relative to the surrounding tissue, we analyzed sections processed for ISH with digoxigenin-labeled probe. We measured the sections' optical density using the luminance feature of Adobe Photoshop (CS2, version 9.0). The luminance values of the gray-scale digital photomicrographs reflect the brightness of a digital image and are inversely related to the optical density of the object imaged. We proceeded in three steps: 1. To account for differences in overall staining intensity between sections, we measured luminance of a relatively large (25–40% of a brain section) unlabeled tissue area and an equivalently sized area with strong label. These values approximated the brightness range of a section and were set to 0 (unlabeled) and 1 (strongly labeled). 2. For each section, we recorded the average luminance value for each of the song nuclei of interest as well as their surrounding area, and normalized these values by to the section's brightness range (calculating in the following way: *normalized_luminance  =  (measured_luminance – non-labeled_reference)/(strongly_labeled_reference – non-labeled_reference)*). 3. To determine the labeling difference between a nucleus and its surroundings, we used the difference between their normalized average brightness values. The procedure was chosen empirically as it was the most robust way to compare sections of different overall staining intensity differences. Comparing the resulting measures across adjacent brain sections yielded comparable results for the same structures (e.g. inside/outside HVC), which was not the case when we used the brightest and the darkest spot in a section.

We determined labeling strength based on the *difference* instead of the *ratio* between nucleus and surroundings to make sure that the values remained related to the overall brightness space covered by the labeling of the section. This means, a labeling difference of 0.1 between a nucleus and its surrounding reflects a brightness difference of approximately 10% of the entire section brightness range, irrespective of whether both regions show strong or weak labeling. We found that using the ratio instead would have resulted in misleadingly high values in cases where both nucleus and surroundings are only weakly labeled.

### Localization of ATRA activity in brain sections using a RARE-LacZ reporter cell assay

The F9-reporter cells which carry a β-gal reporter gene under control of an ATRA-sensitive promoter element [46] were a gift from Prof. Michael Wagner, State University of New York Downstate Medical Center, Brooklyn. F9-reporter cells were grown to subconfluence in 10 cm Petri dishes for subsequent coculture with entire brain slices of 110µm thickness. Growth medium for the cells was Dulbecco's MEM (DMEM; Biochrom) with high glucose content (4.5 mg/ml) and L-glutamine, 20% fetal calf serum (FCS; Biochrom), 1% (v/v) Penicillin/Streptomycin (Roth), and 0.8 mg/ml Geneticin (Gibco). Brains for coculture were obtained from birds overdosed with isoflurane followed by immediate intracardial perfusion with approximately 20 ml PBS and quick dissection. Brains were placed immediately into an 1 1 ice cold mixture of PBS and DMEM medium (4.5 mg/ml glucose, Biochrom), prepared by blending 50% frozen and 50% 4°C cold solutions. Sagittal brain slices of 110µm thickness were cut freshly with a vibratome (Leica). Until transfer to the cell cultures, sections were kept in ice cold medium. Immediately prior to coculture, growth medium in the cell dishes was replaced with a thin layer of assay medium (serum-free, antibiotics-free DMEM medium containing 4.5 mg/ml glucose, Biochrom). Using a brush, brain sections were placed very carefully on top of cell monolayers (typically three to six sections per 10 cm Petri dish) to not destroy the cell layer, and dishes were transferred to a CO_2_ incubator (Binder) for 2.5–3 h for brain slice attachment. Before moving the dishes, medium had to be removed almost completely to prevent sections from dislodging. In the incubator, some drops of medium were added to each dish every 30min to keep sections and cells wet. After 2.5–3 h attaching time additional medium up to 10 ml was carefully added to each dish without moving it. The cocultures were incubated for 24 h, then carefully washed with PBS, fixed with 1% glutaraldehyde (Sigma) in PBS for 15min at room temperature, washed again with PBS and incubated with standard X-gal solution (0.2% X-gal (Roth), 3.3 mM K_3_Fe(CN)_6_ (Roth), 3.3 mM K_4_Fe(CN)_6_ (Roth), 150mM NaCl, 1mM MgCl_2_ in phosphate buffer pH 7.0) at 37°C over one to three nights (duration depended on staining intensity as judged by inspection). After X-gal staining, sections were washed with PBS, coverslipped within the dishes with PBS/50% glycerol (Roth) and stored at 4°C. Photographs of the sections were taken with a Leica Macroscope (MacroFluo Z16APO) through the bottom of the Petri dish to capture the cell layer as well as the sections.

Three controls were run in parallel: (1) F9-reporter cell monolayers not exposed to brain sections but incubated with X-gal (negative control 1), (2) F9-cells without reporter construct cocultured with brain sections and incubated with X-gal (negative control 2), and (3) F9-reporter cells exposed to 5×10^−8^ M ATRA and incubated with X-gal (Sigma; positive control).

### Retrograde labeling of song nuclei

Stereotaxic injections with Alexa-488 conjugated latex beads (Lumafluor) or cholera toxin subunit B conjugates (Molecular Probes) as retrograde neuronal tracers were performed under deep isoflurane anesthesia (1.5–2% isoflurane +2.5l O_2_/min). The birds' heads were placed in a stereotaxic apparatus (MyNeurolab) and the retrograde tracer was injected into song nucleus RA (stereotaxic coordinates, relative to the 0-point at the bifurcation of the midsagittal sinus: medial/lateral 2.4 mm, anterior/posterior −1.8 and −1.5 mm, dorsal/ventral −2.0 and −1.8 mm; injection needle tilted in anterior/posterior plane by -0.9 mm) with a hydraulic micromanipulator (Narishige). Per injection site, approximately 200 nl of tracer were injected. Birds received painkiller orally (Meloxidyl; active ingredient meloxicam, dose 0.1 mg/kg) 30min before anesthesia, and once per day for three days post-surgery. Directly after the surgery, they are monitored for pain or unusual behavior hourly for 5 h, and four times a day for the following two days. Birds were allowed to survive for at least four more days and then killed by decapitation after an overdose of isoflurane. Brains were quickly dissected, hemispheres were separated and frozen immediately over liquid nitrogen and stored at −75°C until processed. Sections were cut with a cryostat (Leica) in the sagittal plane at 14µm thickness. For immunohistochemistry, birds were perfused intracardially with PBS followed by 4% paraformaldehyde (PFA; Sigma), their brains were dissected, post-fixed in 4% PFA overnight and stored in PBS at 4°C until cut with a vibratome (VT 1000S, Leica).

### Lesion surgeries

Birds were administered a pain killer (Meloxidyl; active ingredient meloxicam, dose 0.1 mg/kg), anesthetized with isoflurane, and placed in a stereotaxic apparatus as for tracer injections. For HVC lesions, the skull and the hippocampus overlying HVC were opened using an injection needle to expose HVC. HVC was then removed with the help of a Delicate Bone Scraper (Fine Science Tools). The removed area was oval-shaped with its tip pointing rostrally and its main axis forming a 45-degree angle with the midline. Coordinates were medial/lateral 3.0 mm and anterior/posterior 1.8 mm; medial/lateral 0.9 and anterior/posterior -0.2 mm. Dorsoventral depth of the lesion was about 1 mm. For lesions of the fiber tract between HVC and RA, a knife cut was made about 1.5 mm ventrally to the posterior end of HVC as estimated with the above coordinates and extended about 2 mm into the tissue, about as wide laterally as covered by the above coordinates for HVC. We cannot rule out the possibility that this cut may have damaged fibers running from lMAN to RA as well. After surgery, birds survived 14 days before being sacrificed (followed by PBS perfusion if brains were to be used for reporter assay, or PBS/PFA perfusion for immunohistochemistry).

### Immunohistochemistry

Male zebra finches were perfused intracardially, and brains were cut sagittally into 40 to 60µm sections with a vibratome (Leica). Sections were heat-treated to 95°C for 30min in a 10 mM sodium citrate buffer, pH 6.0 (Fluka). Sections were then incubated with a goat anti-Human ALDH1A2 antibody (sc-22591, 1 50, Santa Cruz Biotechnology) raised against a region near the N-terminus of the human protein (human ALDH1A2, mouse RalDH2 and zebra finch zRalDH are homologous); the secondary antibody was mouse anti-goat biotinylated (1 200, Vector Laboratories). Incubations were performed overnight at 4°C with the primary and for 2 h at RT with the secondary antibody, and followed by several washes withPBS. Sections were developed with the avidin-biotin peroxidase method (Vector Laboratories) using diaminobenzidine (DAB, Sigma-Aldrich) as a substrate, and counterstained with DAPI (4'-6-Diamidino-2-phenylindole; Serva). As a specificity control, additional sections were reacted in parallel with the same antibody pre-incubated with an ALDH1A2 immunizing peptide (Santa Cruz Biotechnology), twice as concentrated as the antibody; no staining was observed (fig. S3).

## Results

### Identification of zebra finch orthologs of Retinoid X Receptors (RXRs)

In mammals, 3 distinct RXR genes have been described, RXRα, β, and γ [47]. In order to identify zebra finch RXR orthologs, we searched the zebra finch genome using BLAT alignments as well as extensive blast searches of zebra finch EST/cDNA databases, using orthologous chicken, mouse, and human sequences as queries. We also compared the syntenic groups in zebra finch with those in chicken, the green anole lizard and mammals. In contrast to mammals, we could only identify RXRα and γ in birds. As in chicken, the zebra finch RXRα gene is located on chromosome 17, similar to chicken and mammals, and it is flanked by the Col5a1 and Wdr5b genes. Also similar to chicken, the RXRγ gene resides on chromosome 8 and is flanked by the Sell and Lmx1a gene. This synteny is only partially shared with mammals, where the RXRγ gene is flanked by Aldh9a1 and Lmx1a. Neither chicken nor zebra finch genomes seem to contain an RXRβ gene, in fact the entire syntenic region containing this gene seems to be absent in birds (data to be published in a separate study). The RXRβ gene is present in lizard and turtle, suggesting that the loss of this gene was in the avian lineage.

### Expression of RXRα and RXRγ in the adult zebra finch brain

**Figure 3:**
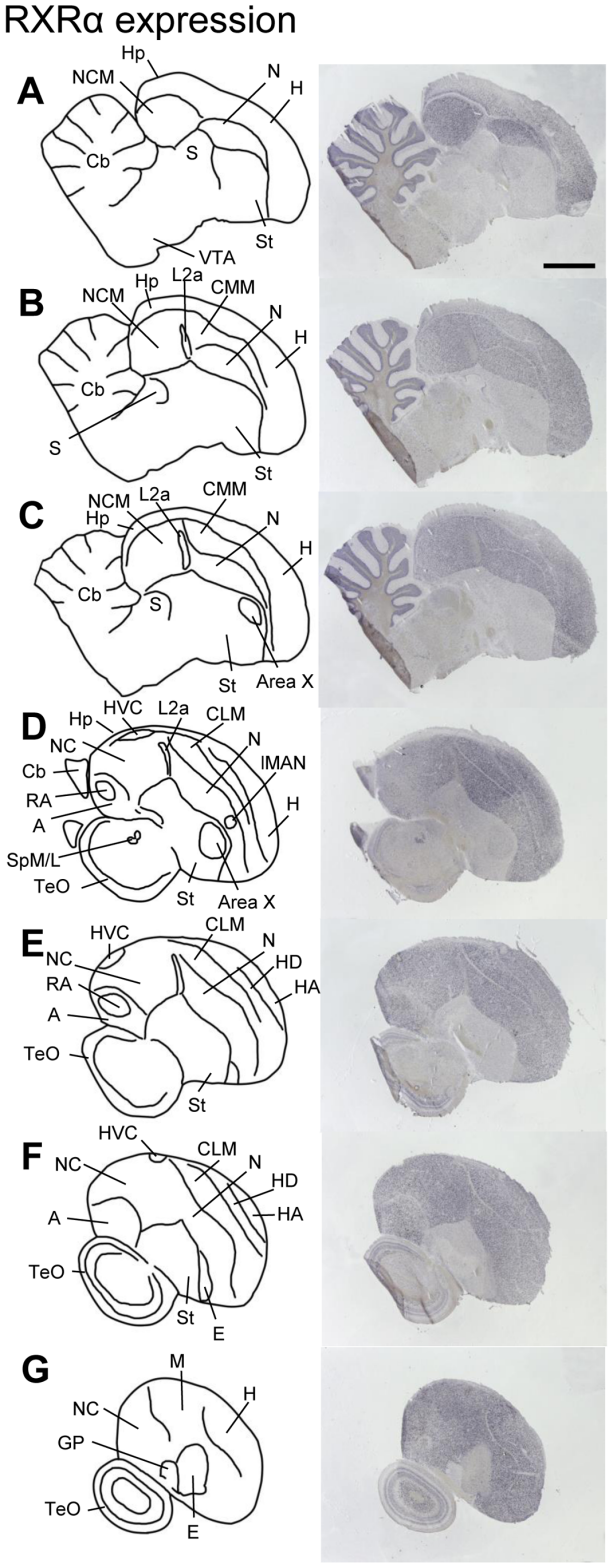
RXRα expression in adult male zebra finch brain. Drawings on the left depict brain areas and nuclei of serial parasagittal sections. The corresponding sections on the right were processed for *in situ* hybridization (ISH) for RXRα. For all images, anterior is to the right and dorsal is up; medial to lateral levels are represented from top to bottom. Scale bar  = 2 mm (all panels). For abbreviations, see table 1.

In adult males, RXRα expression was widespread, resembling the distributions of RARα, β and γ [29] rather than the more restricted expression of zRalDH [21], [25], [26]. All pallial regions (hyperpallium, mesopallium, nidopallium, and arcopallium) expressed moderate and uniform levels of RXRα, whereas expression was lower in the hippocampal formation, and very low in the striatum, globus pallidus, entopallium and Field L2a (fig. 3). Expression in the thalamus, hypothalamus, midbrain, pons, and medulla was low to non-detectable, except for both subdivisions of the nucleus spiriformis (fig. 3.D and fig. S4) and the dopaminergic VTA/SN regions (fig. 3.A and fig. S4). In the cerebellum, the granule cell layer expressed RXRα (fig. 3.A–C), whereas labeling was absent in the molecular layer.

**Figure 4:**
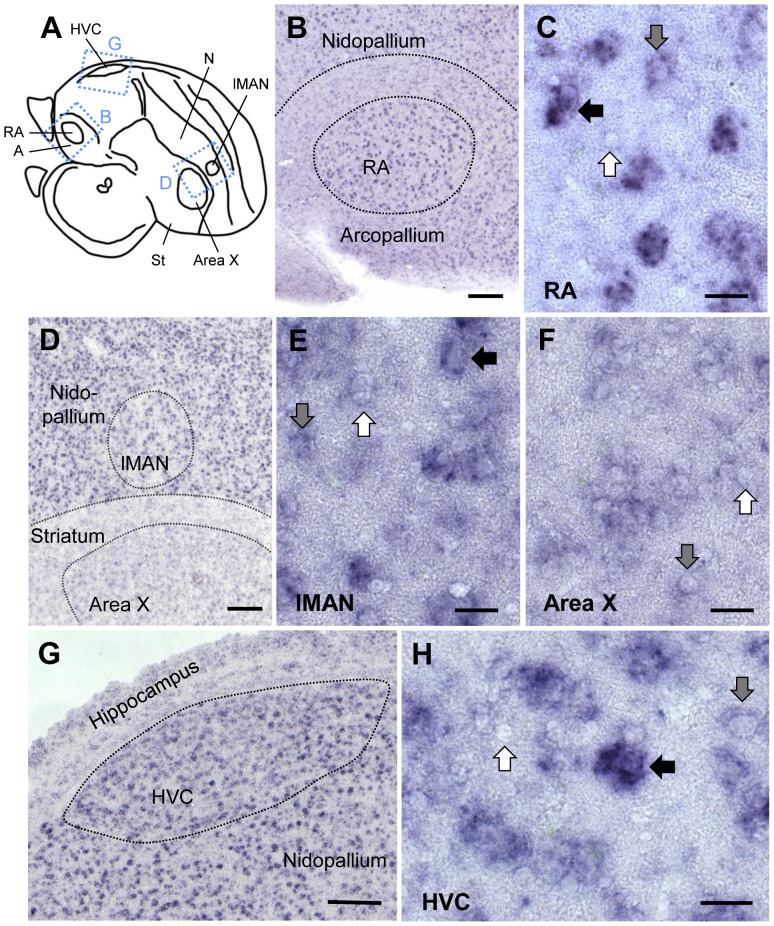
RXRα expression in song control nuclei of adult male zebra finch. A: Schematic of a parasagittal section at the level of song nuclei HVC, RA, Area X, and lMAN. The dashed rectangles indicate the areas shown in B, D, and G. Photos in B–H are bright field views of parasagittal sections hybridized with digoxigenin-labeled RXRα antisense probe. Anterior is right, dorsal is up in all panels. B, C: Expression in RA. Many cells were strongly labeled; the surrounding arcopallium showed less labeling. D-F: Expression in lMAN and Area X. Expression is comparable to the surrounding areas, but lower in Area X/striatum than in lMAN/nidopallium. G, H: Expression in HVC. The high expression is comparable to the adjacent nidopallium. In C, E, F, and H, black arrows depict strongly labeled cells, gray arrows weakly labeled cells, and white arrows unlabeled cells. Scale bars  = 200µm in B, D, G; 20µm in C, E, F, H.

RXRα expression was moderately high in pallial HVC, RA, and lMAN and very low in striatal Area X (fig. 4). Within HVC, most strongly labeled cells were large with a neuron-like shape, but cells with different degrees of labeling, as well as non-labeled cells, were found as well (fig. 4.G,H). In RA, many cells showed high expression, but weakly or unlabeled cells also existed (fig. 4.B,C). In lMAN, only the large cells were strongly labeled, the several unlabeled smaller cells contributing to the apparent lower overall expression than in the adjacent nidopallium. (fig. 4.D,E). Area X lacked strongly labeled cells altogether; instead, many weakly labeled and non-labeled cells were seen (fig. 4.F). Staining in RA differed markedly from its surrounds, where labeling was weaker (fig. 4.B and 3.D,E), while staining was more similar between Area X, lMAN, HVC, and their surrounds (fig. 4.D,G). lMAN expression differed markedly across individuals, ranging from distinctively lower to comparable to the surrounding nidopallium (fig. 4.D, for further analysis of expression variability in lMAN, see below).

**Figure 5:**
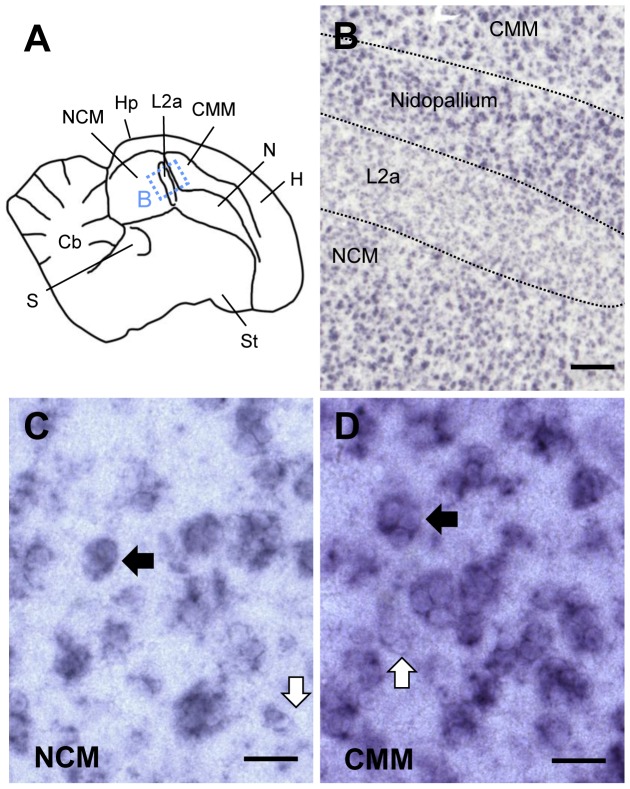
RXRα expression in higher auditory areas of adult male zebra finch telencephalon. A: Drawing of a parasagittal section at the level of the auditory areas NCM, Field L2a, and CMM. The dashed rectangle indicates the area shown in B. B–D: Bright-field views of parasagittal sections processed for RXRα *in situ* hybridization. Anterior is right, dorsal is up in all panels. B: Low power view of higher auditory areas, with strong labeling in NCM and CMM and lower labeling in L2a. C, D: High power views of NCM and CMM, respectively; black arrows depict strongly labeled cells in clusters, white arrows depict unlabeled cells. Scale bars  = 200µm in B; 20µm in C, D.

RXRα expression was also high in auditory NCM and CMM; fig. 5.A,B), in contrast to the low expression in the adjacent core thalamorecipient field L2a (fig. 5.B) and in the overlying hippocampus. Numerous high RXRα-expressing cells occurred throughout NCM and CMM (fig. 5.C,D), although negative cells were also present (see fig. 5.C,D; white arrows).

**Figure 6:**
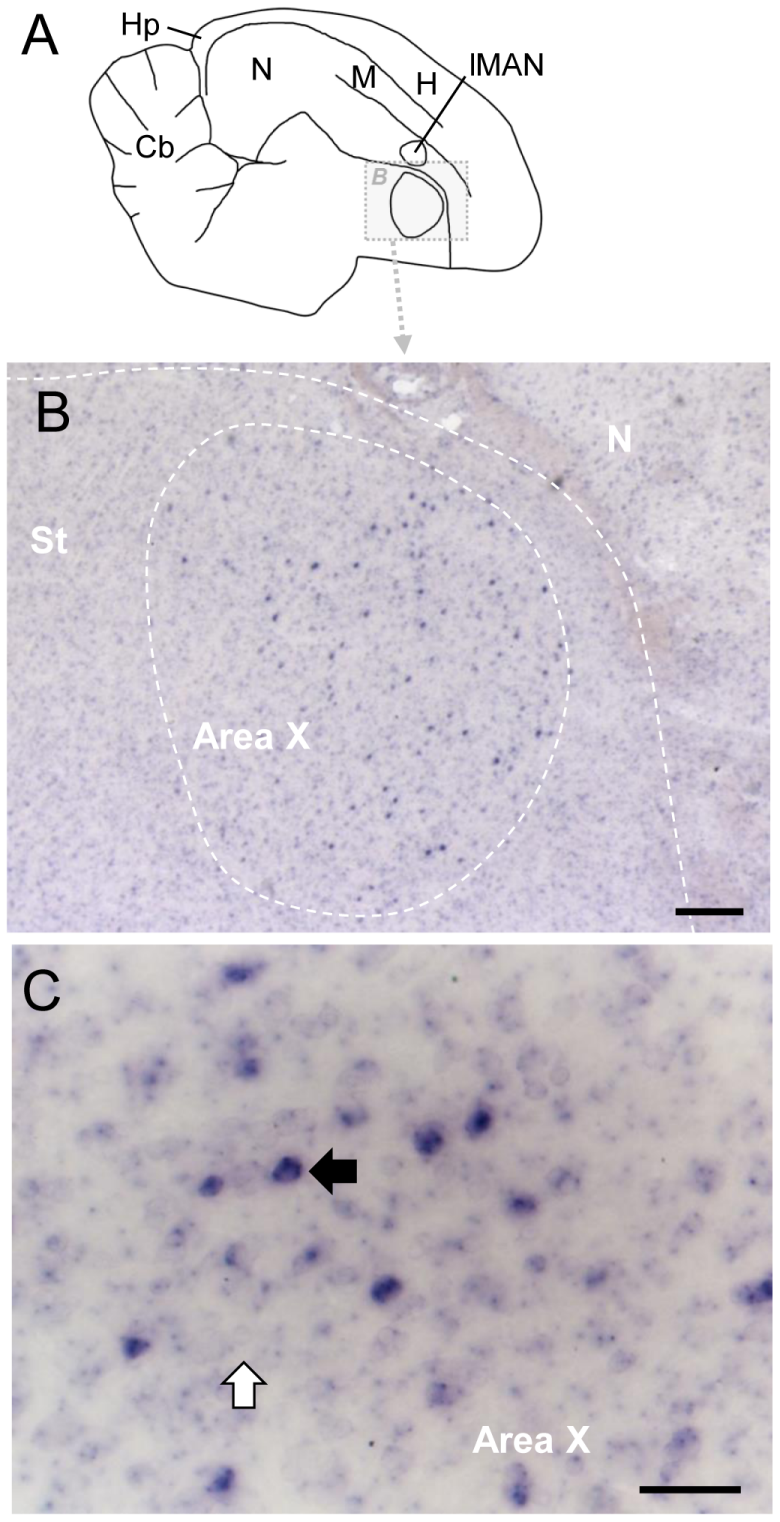
RXRγ expression in Area X of adult male zebra finch. A: Drawing of a parasagittal section of adult brain, indicating detail area shown in B; anterior is to the right, dorsal is up. For abbreviations see table 1. B: Detail view of Area X and surrounding area in section processed for RXRγ ISH showing sparse labeled cells in Area X. D: High-magnification view of Area X; black and white arrows depict labeled and unlabeled cells, respectively. Scale bars: 2mm in B, 200µm in C, 50µm in D.

RXRγ expression was low and could not be visualized with phosphorimager autoradiography. However, high-power examination of sections reacted with digoxigenin-labeled riboprobes revealed expression in a discrete cell population within Area X (fig. 6); no other RXRγ positive cells were found in the subpallium or pallium. These RXRγ positive cells were large and sparsely distributed (fig. 6.B,C). The same pattern was found in juvenile males (42 and 64 days old; not shown).

### Regions of ATRA localization in the zebra finch brain as determined by a reporter assay

**Figure 7:**
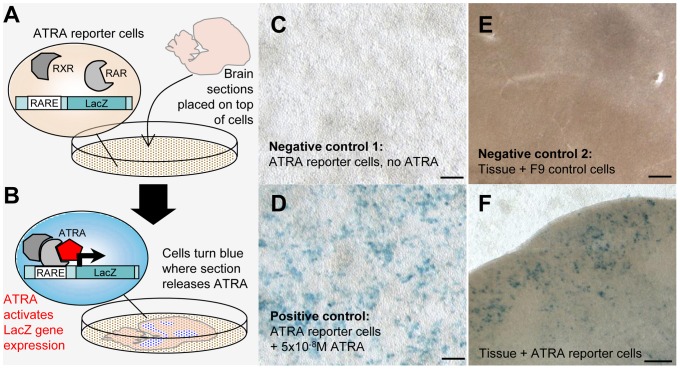
ATRA mapping in brain sections through a reporter cell assay. A and B: Schematic overview of ATRA reporter cell assay. A: Reporter cells (they contain a LacZ gene under a retinoic acid response element, or RARE, and express retinoic acid and retinoid X receptors - RAR/RXRs - needed for ATRA induced gene expression) are seeded onto a Petri dish; a freshly cut brain section is placed into the dish and attaches to the cell monolayer. B: ATRA locally generated in the brain slice reaches a reporter cell, binds to RAR/RXR complexes and causes LacZ expression, revealed as blue label by LacZ/X-gal staining. C and D: LacZ expression in reporter cells is specifically induced by ATRA. C (negative control 1): In the absence of ATRA, reporter cells are LacZ-negative and do not turn blue upon LacZ/X-gal staining. D (positive control): Labeling is generated when ATRA is added to the medium. E (negative control 2): A slice co-cultured with an F9 cell line *without* RARE and LacZ does not generate blue signal upon LacZ/X-gal staining. F: A slice from an adult male bird co-cultured with reporter cells results in blue labeling in regions where ATRA is present. Blue labeling is seen under song nucleus HVC, which expresses zRalDH, but not in cells without overlying tissue (top), or in cells that underlie a part of the tissue that does not contain ATRA (bottom). Photos in C–F were taken through the bottom of the Petri dish. Scale bars: for C, D  = 200µm; for E, F = 100µm.

To localize sites of ATRA presence in brain sections, we used a reporter cell assay (fig. 7.A,B) consisting of a monolayer of mouse F9 cells carrying a LacZ reporter under the control of a retinoic acid response element (RARE). ATRA produced by tissue samples placed on top of the reporter cell layer induces LacZ expression [46], [48]. To detect ATRA-induced gene expression in entire brain sections, we used a modified assay (details in fig. 7.C–E, Methods, and table S2). We observed spatially specific LacZ expression in all birds (fig. 7.F). Reporter cells that were not touched by the co-cultured tissue sections did not show any blue LacZ staining, even if located directly adjacent to the edge of tissue that generated staining in the cell monolayer (fig. 7.F). In addition, boundaries between tissue regions that expressed LacZ and those that did not could be relatively sharp, as was the case for some regions close to HVC (fig. 7.F), indicating that ATRA did not freely diffuse across the reporter cell monolayer, but reflects sites of ATRA presence in the brain sections.

**Figure 8:**
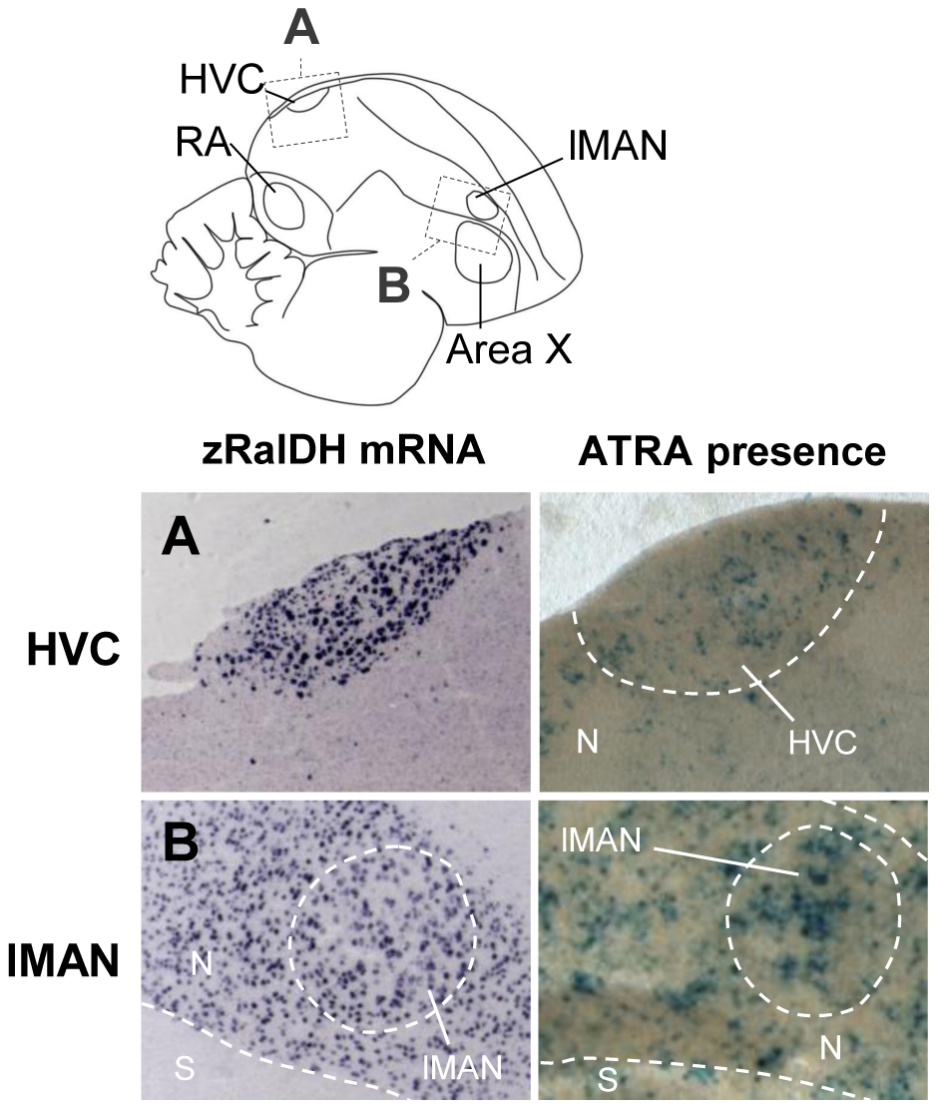
ATRA-induced reporter expression in song nuclei HVC and RA of adult male zebra finch is consistent with zRalDH expression. The drawing illustrates regions shown in A (HVC) and B (lMAN), abbreviations in table 1. Left panels show ISH for zRalDH, right panels show detection of ATRA (blue label) in brain sections by reporter assay. HVC but not the surrounds, and lMAN and its surrounds both strongly express zRalDH and induce reporter expression. In all panels, frontal is to the right and dorsal is up.

**Figure 9:**
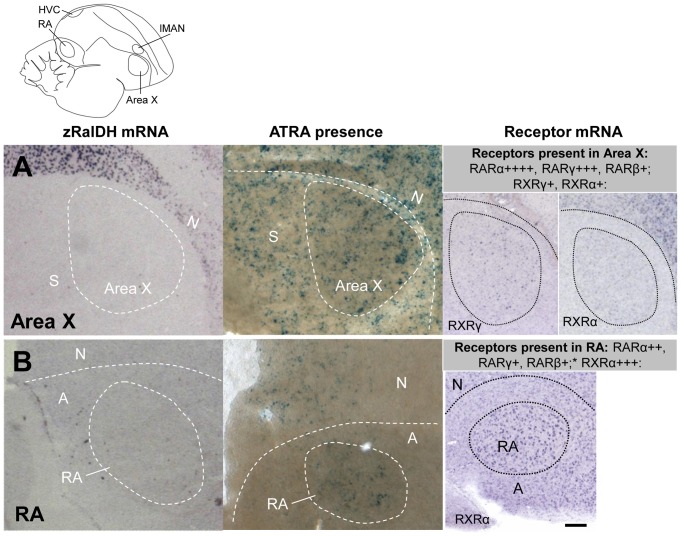
Induction of ATRA reporter expression by song nuclei that do not express zRalDH in adult male zebra finch. Line drawing on top indicates position of song nuclei. Left panels: detail views of zRalDH expression by ISH. Middle panels: Sites of ATRA presence by reporter cell assay. Right panels: summary and examples of transcript distribution for retinoid receptors. Data for RAR expression are from Jeong et al., 2005. Brain diagram on top indicates position of song control nuclei. In all panels, frontal is to the right and dorsal is up. A: Area X; B: RA. In both nuclei, zRalDH is not expressed, but ATRA-induced reporter expression is detected, as well as receptors that may mediate ATRA effects.

We detected ATRA-induced LacZ expression in all brain areas that express zRalDH, including HVC and lMAN (fig. 8), confirming previous results [21]. Interestingly, ATRA also induced LacZ expression in some regions distant from zRalDH expression sites, such as Area X and RA (fig. 9). It seems unlikely that the RA and Area X signal was due to diffusion since there was less LacZ expression in the arcopallial tissue surrounding RA than inside the nucleus (fig. 9.B), which would not be the case if ATRA had diffused from around RA into the nucleus. Furthermore, the closest zRalDH mRNA expression sites, lMAN and its surrounding nidopallium, are too distant to account for the observed distribution of ATRA-induced gene expression (fig. 9).

### Distribution of zRalDH protein in the zebra finch brain

**Figure 10:**
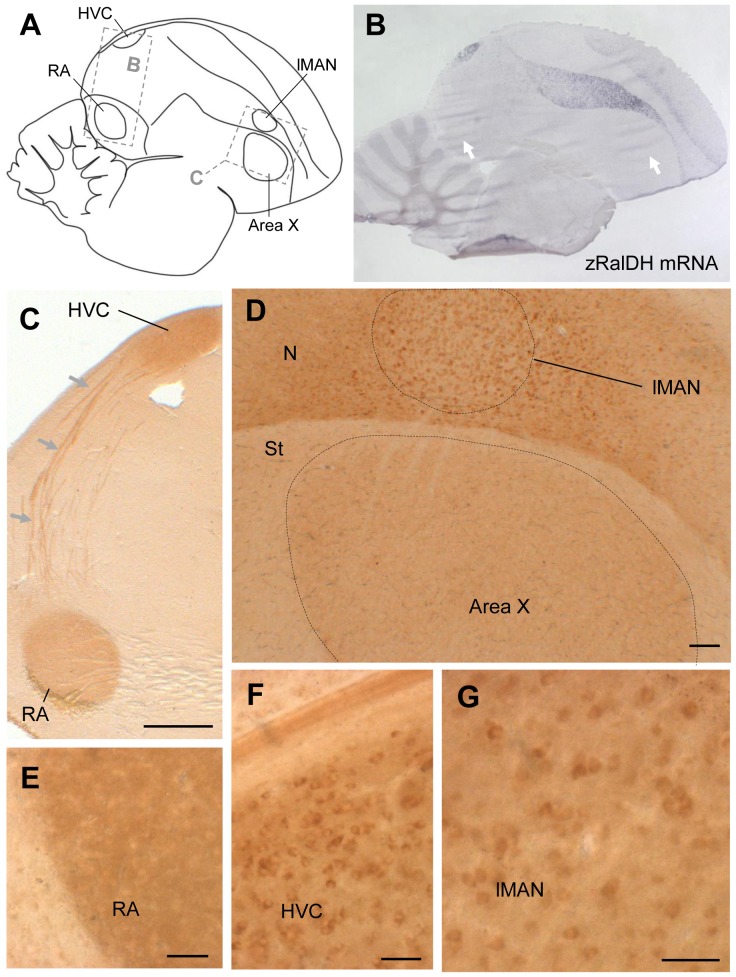
zRalDH protein expression in areas of adult male zebra finch brain that express or lack zRalDH mRNA. A: Drawing indicates location of images shown in other panels. B: zRalDH mRNA expression by *in situ* hybridization, level similar to A. C–G: zRalDH protein detection through immunohistochemistry. C: HVC and RA, as well as the fiber tracts extending from HVC to RA (arrows) are labeled. D: lMAN and surrounding nidopallium, as well as Area X, are labeled; labeling in Area X is diffuse and not in somata. Thus, besides HVC and lMAN, which express zRalDH mRNA, protein is present in two song nuclei (X and RA) that lack zRalDH transcript (white arrows in B). E: Detail view of zRalDH protein in RA; labeling is diffuse and not in somata. F: Detail view of zRalDH protein in HVC, somata are labeled. G: High power view of zRalDH in lMAN, somata are labeled. In all panels, frontal is to the right, and dorsal is up. For abbreviations, see table 1.Scale bars for C = 0.5 mm; D–F = 100µm, G = 50µm.

To examine whether transport of zRalDH protein to brain sites distant from transcription sites could account for the broad distribution of ATRA-induced LacZ expression, we localized zRalDH protein using immunohistochemistry (fig. S3). Table S3 summarizes the birds used for zRalDH immunohistochemistry, and the treatments they underwent. We detected immunoreactivity not only in all brain areas that expressed the zRalDH transcript (i.e. the rostral nidopallium including lMAN, the hyperpallium, and HVC) but also in Area X and RA (fig. 10).

**Figure 11:**
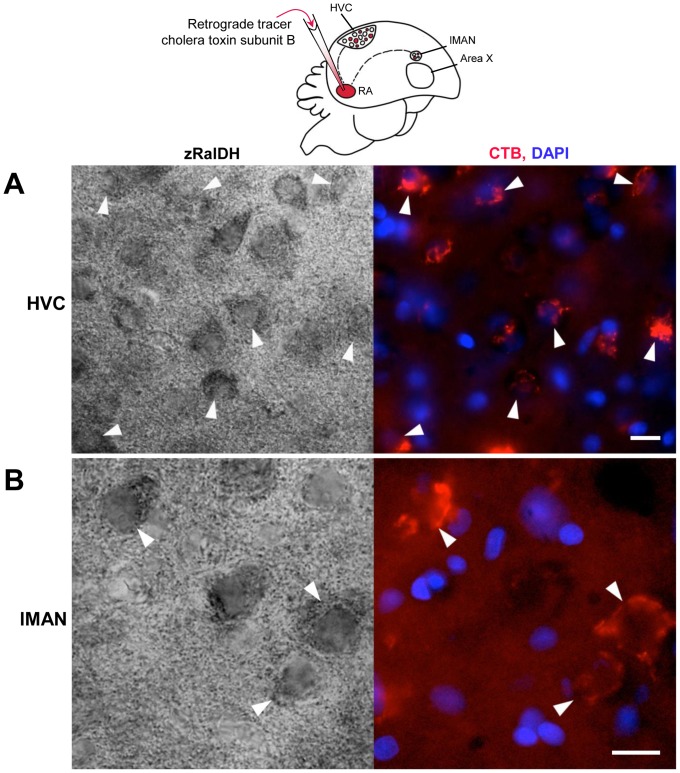
zRalDH immunoreactivity in HVC and lMAN neurons that project to RA (HVC_RA_ and LMAN_RA_). Schematic drawing on top illustrates injection of the retrograde tracer cholera toxin subunit B (CTB) and location of retrogradely labeled neurons. A and B: High power views of HVC (A) and lMAN (B) in zRalDH-immunolabeled sections from an adult male zebra finch that received an CTB injection into RA resulting in retrogradely labeled neurons in HVC and lMAN. Left panels show bright field views of zRalDH protein expression, right panels show CTB signal (red) and cell nuclei stained with DAPI (blue) in the same fields. Arrowheads point to zRalDH-immunoreactive cells that are retrogradely labeled. Scale bars: 20µm.

A close look revealed that while zRalDH immunolabeling in HVC and lMAN was concentrated in cell somata (fig. 10.F,G), it was diffuse in RA (fig. 10.E), a pattern consistent with zRalDH distribution at synaptic endings of afferent projection fibers. When we combined zRalDH immunohistochemistry with retrograde tracer injections into RA, zRalDH-labeled cells in HVC and lMAN co-localized with the retrogradely-labeled cell somata in these nuclei, corresponding to HVC_RA_ and lMAN_RA_ projection neurons (fig. 11). Area X also exhibited a diffuse zRalDH immunostaining (fig. 10.D), which could stem from either lMAN or HVC. In the case of Area X, however, no zRalDH positive fiber tracts were visible (fig. 10.D).

### Identifying the origins of the ATRA present in RA

**Figure 12:**
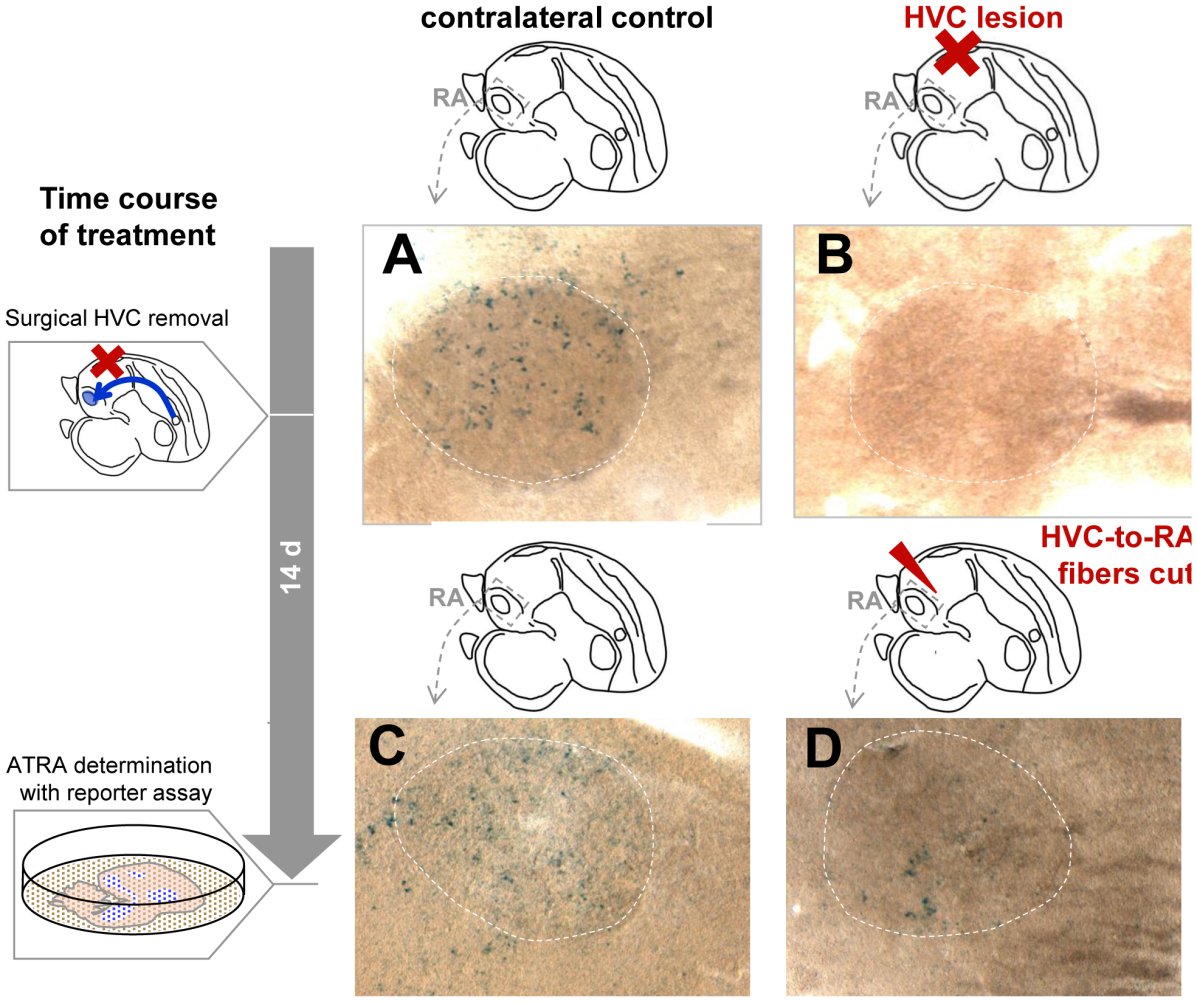
In the absence of axonal input from HVC, ATRA induced reporter staining is reduced in RA of adult male zebra finches. Experimental design and time course are indicated by diagrams on the left and on top of photomicrographs. After surgical procedure, birds were allowed to survive for 14 days before reporter assay was performed. A–D: High power views of reporter expression induced in the monolayer by RA; dashed circles indicate position of RA. A and C: control hemispheres with intact HVC-to-RA projections. B and D: experimental hemispheres with HVC lesion (B) or knife-cut fibers (D).

To determine whether terminal fibers from HVC projection neurons might be the source of the ATRA that induced LacZ expression in RA, we performed unilateral lesion ablations of HVC in 7 male zebra finches (3 adults and 2 juveniles) 14 days before subjecting their brain tissue to the ATRA reporter cell assay. In the absence of axonal input from HVC, ATRA-induced LacZ was strongly reduced or undetectable in RA (fig. 12.A,C). A similar result was obtained in 2 additional adult males where, instead of removing HVC, the fiber tracts from HVC to RA were cut with a scalpel 14 days before sacrifice (fig. 12.B,D). This procedure may have also affected fibers from lMAN to RA, as these travel in parallel to the HVC_RA_ fibers. Even though the reporter assay used only allows a qualitative assessment of labeling intensity, it can be inferred from the very weak LacZ staining (specifically, very few to no cells in the reporter monolayer retained staining) that ATRA in RA after HVC removal or fiber cut was low to non-detectable. These findings, together with the diffuse zRalDH immunoreactivity seen in RA, suggest that ATRA in RA stems from outside sources; while HVC seems to be a major source of zRalDH enzyme/ATRA in RA, we cannot discard the possibility from these current experiments that LMAN is also a source.

### Dynamic RXRα expression: age and time of day influence expression in lMAN

**Figure 13:**
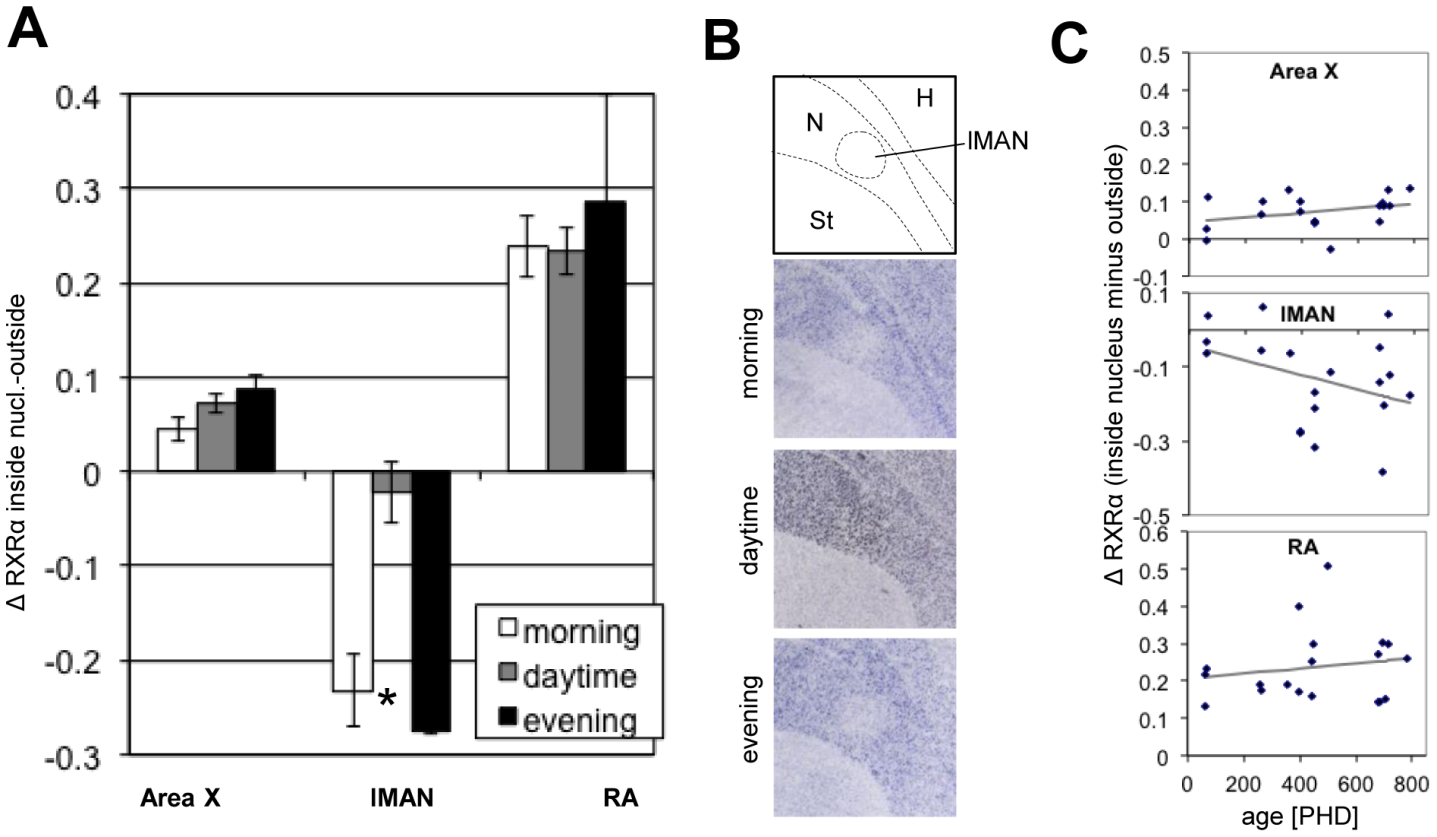
RXRα expression in Area X, lMAN, and RA in relation to time of day and age. A: RXRα expression across the day. Bars represent the difference in normalized expression strength between a song nucleus and the surrounding area (“Δ RXRα inside nucleus-outside”, mean ± SEM); a 0 value indicates no expression difference, positive values indicate more expression inside than outside, negative values the opposite. At different times, expression compared to the surrounds varied significantly in lMAN, but not in Area X or RA; asterisk indicates significance by MANCOVA (Area X: F = 0.79, p = 0.471; lMAN: F = 5.7, p = 0.014; RA: F = 0.31, p = 0.736); for details, see Methods. During the day, the RXRα expression inside and outside lMAN was similar, whereas RXRα expression was lower in lMAN than in the surrounding nidopallium in the morning and evening. B: Representative bright field images of sagittal sections showing variation in RXRα expression in lMAN compared to surrounding nidopallium at different times of the day. C: RXRα expression across ages. Plotted is the same measure of expression strength as in A vs. age (post-hatch days), and linear fits; a significant decrease with age was seen in lMAN (MANCOVA, F = 4.72, p = 0.046) but not in Area X (F = 2.13, p = 0.165) or RA (F = 0.54, p = 0.476).

Because of the known influence of retinoid signaling on circadian and seasonal rhythms in mammals [49] we explored whether RXRα expression in the song system varied with time of day, as well as age. Birds of different ages were sampled at different times of the day (without sampling at all times of the day for each age) and statistical significance for effects of age and time of the day were determined using MANCOVA (for details, see Materials & Methods). Compared to the adjacent tissue, RXRα mRNA expression varied with both age and time of the day in lMAN, but not in HVC, RA, or Area X (fig. 13). During the day (i.e. from 2 h after lights turned on until 2 h before lights turn off in a 12 12 h light cycle), RXRα expression was comparable between lMAN and surrounds (n = 14), whereas in the morning (within 20min after the lights turned on, before any singing took place; n = 3) and in the evening (within the last 1.5 h before the lights turned off, n = 2), RXRα expression was lower in lMAN than in the surrounding nidopallium (fig. 13.A,B). In adults, lMAN expressed less RXRα than the surrounding nidopallium. This difference was significantly less pronounced in juveniles (fig. 13.C).

Interestingly, inspection of zRalDH immunopositive material suggested that there may exist an age-related decline of zRalDH expression as well (fig. S5): Similar to RXRα, zRalDH seems to be dynamically regulated within lMAN. The older the animal, the fewer cells per area seemed to conspicuously express zRalDH, while the expression strength per zRalDH positive cell did not appear to strongly vary with age (fig. S5).

### Expression of retinoid degrading enzymes in higher order auditory areas

**Figure 14:**
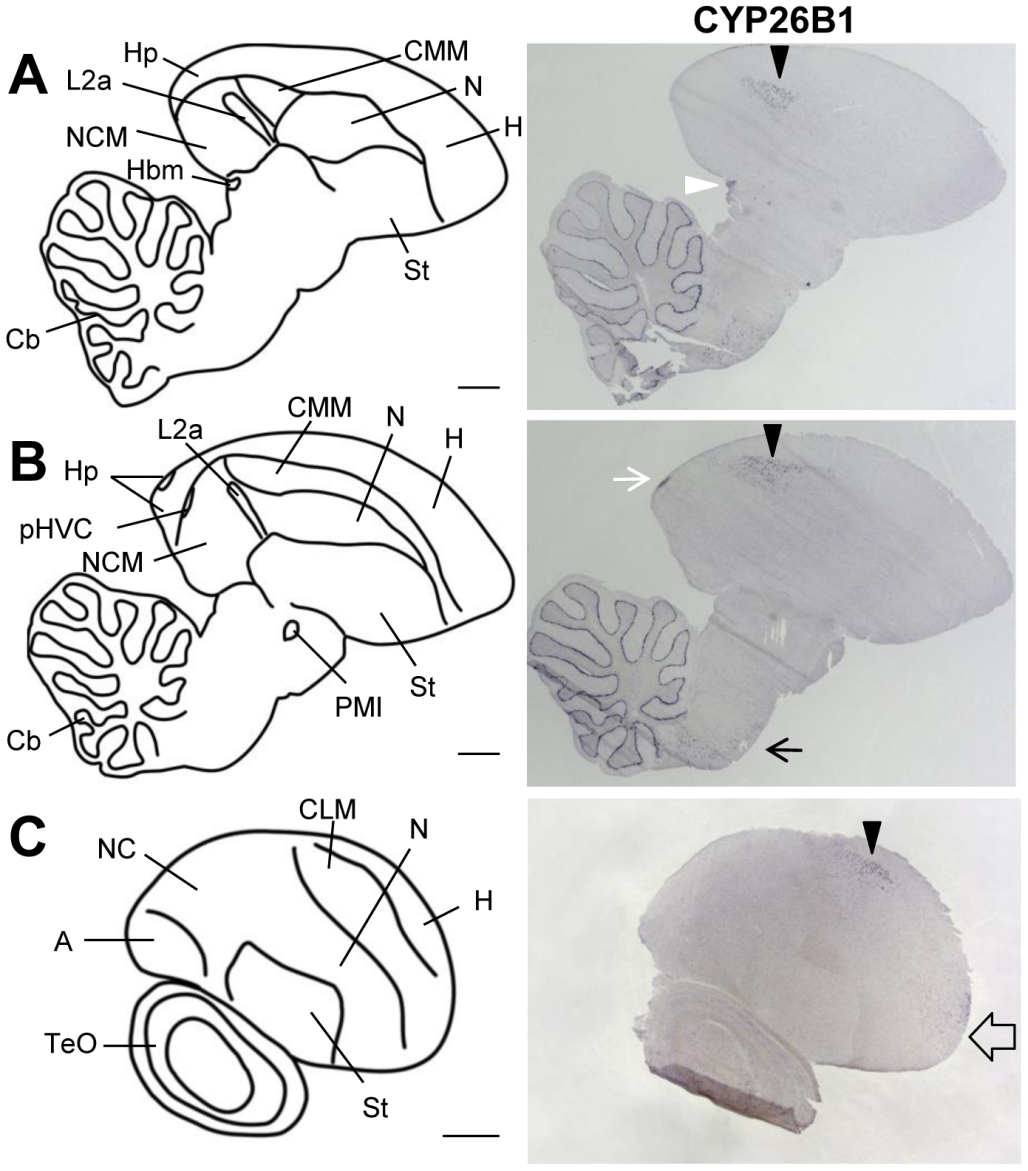
CYP26B1 expression in adult zebra finch brain. The drawings on the left depict brain areas of the parasagittal sections on the right. The right panels show sections processed for ISH for CYP26B1. For all images, anterior is to the right and dorsal is up. A and B are medial sections, C a lateral one. CYP26B1 expression is sparse, mostly in CMM and CLM (black arrowheads), and does not overlap with zRalDH (see fig. 14, 15, and fig. 7). CYP26B1 is also expressed in the fronto-lateral mesopallium (empty arrow), in the medial habenula (white arrowhead), in the caudal-dorsal hippocampus (white arrow), and in cerebellar Purkinje cells. For abbreviations, see table 1. Scale bars  = 1 mm.

**Figure 15:**
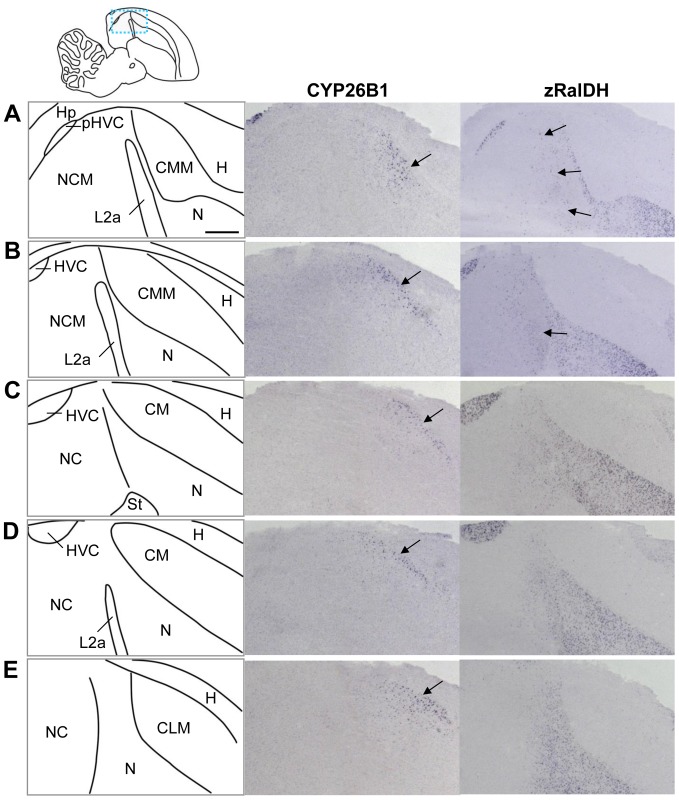
CYP26B1 and zRalDH exhibit graded expression in higher auditory areas of adult zebra finch. The drawing in the top left indicates approximate regions shown in A–E; the drawings on the left depict brain areas shown on the right. The middle and right columns show sections processed for ISH for CYP26B1 and zRalDH. For all images, anterior is to the right and dorsal is up; medial to lateral is represented from top to bottom. CYP26B1 is expressed in a dorsoventral gradient-like pattern throughout the caudal, caudo-medial and caudo-lateral mesopallium (CM, CMM and CLM; arrows in middle panels point to region of high expression). This distribution does not overlap with zRalDH mRNA expression, which is absent in the mesopallium; some cells in the rostral part of the caudal nidopallium express zRalDH (arrows in right panels), tapering off caudally. For other abbreviations, see table 1. Scale bar  = 0.5 mm.

We mapped the mRNA distribution of the retinoic acid degrading cytochromes CYP26A1, CYP26B1, and CYP26C1 in juvenile (6 males, PHD 20-68) and adult (7 males, 2 females) zebra finches. CYP26A1 and CYP26C1 distributions were low, broad and uniform, without evidence of regional enrichments (data not shown). In contrast, we successfully detected strong labeling at the known sites of CYP26A1 and CYP26C1 expression on sections of a zebra finch embryo (data not shown), demonstrating the effectiveness of the probes. CYP26B1 showed a complex pattern (fig. 14) that was partly adjacent to, but did not overlap with zRalDH expression. Within the telencephalon, CYP26B1 expression was restricted to the caudal mesopallium (CM), with highest expression in the dorsal-most part (fig. 14 and fig. 15) but some expression in the most fronto-lateral part (fig. 14.C), and a very small region in the hippocampus (fig. 14 and fig. S6.B–E). Outside of the telencephalon CYP26B1 was expressed in the medial habenula (fig. S6.F–H), the Purkinje cell layer of the cerebellum (fig. 14.A,B), and some scattered cells in the brain stem (fig. 14.B). Comparing distributions of the transcripts for the ATRA synthesizing zRalDH and the ATRA degrading CYP26B1 reveals differential expression combinations across the higher auditory regions (fig. 15): In CM (including both CMM and CLM), CYP26B1 expression varied along the dorso-ventral axis in a gradient-like manner, while zRalDH transcript was entirely absent. In NCM, which is devoid of CYP26B1 transcript, we noted some zRalDH expression in scattered cells concentrating in the most anterior part, while no zRalDH positive cells are found in the more posterior NCM (fig. 15.A–C). Note that this is in contrast to a previous study [25], where NCM did not show any zRalDH expression at all. CMM and NCM are separated by a thin layer of caudal nidopallium with high zRalDH expression, and field L2a which did not express either of the genes (fig. 15).

The distributions of CYP26B1 and zRalDH transcripts raise the possibility that ATRA is distributed differentially across higher auditory areas, apparently in a gradient-like manner. Indeed, the ATRA reporter cell assay data are consistent with this hypothesis, showing high staining in the anterior mesopallium, and decreasing towards its caudal and dorsal part (fig. S7). Mesopallial ATRA may result from diffusion from the adjacent zRalDH expressing hyper- or nidopallium, as zRalDH is not present in mesopallium, or from synthesis by a different ATRA generating enzyme. The lack of ATRA-induced reporter expression in the dorso-caudal mesopallium was consistent with expression of CYP26B1 in the same region, suggesting that CYP26B1 may create a local ATRA sink. Consistent with this idea, CYP26B1 expression decreased towards the rostro-ventral mesopallium, whereas ATRA-induced LacZ staining increased.

Receptor expression patterns in the higher auditory areas are in line with complex ATRA signaling. RXRα expression was high throughout NCM and CM, with strongest expression in NCM (fig. 3 and fig. 5), and all three RARs are expressed in both NCM and CMM to different degrees and with differential distributions [29]. Field L2a which is located between those two areas is largely devoid of expression of any retinoid related gene (fig. 3, fig. 5, fig. 6, fig. 14, and study by Jeong et al. [29], for RARs).

Brains of two adult females and six juvenile males (PHD 20–68) showed comparable expression patterns as the adult males reported above (data not shown).

## Discussion

### Distribution of retinoic acid pathway genes in the adult song control system: General overview

Our data mapping the distribution of RXRα, RXRγ, CYP26B1, ATRA, and zRalDH in the zebra finch brain indicate a previously unrecognized complexity of the retinoic acid signaling system [21], [29], [50]. Specifically, we found that ATRA is able to induce reporter gene expression in two song nuclei, RA and Area X, that do not express the retinoic acid synthesizing enzyme zRalDH. Because we detected zRalDH protein in HVC neurons that project to RA and to Area X, as well as in lMAN neurons that project to RA and Area X, we propose that ATRA in Area X and RA originates in HVC and/or lMAN. Consistent with this notion, immunoreactivity for zRalDH protein could be detected along the fibers bundles where the fibers of the projection pathway from HVC to RA are located and immunolabeling in the target nuclei was diffuse, suggesting the protein is present in presynaptic endings. More importantly, surgically disconnecting HVC and lMAN from their targets led to reduced ATRA-induced reporter expression in Area X and RA.

Finding that zRalDH enzyme can apparently be transported between song nuclei, and detecting ATRA presence in both source and target areas, resolves a puzzling discrepancy between zRalDH and RAR mRNA distributions: zRalDH mRNA is strictly confined to few brain areas including HVC, lMAN, and a nidopallial gradient around lMAN [21], while RAR expression is much more widespread [29]. RAR mRNA is found at sites far away from zRalDH expression areas, including song nuclei RA and Area X. We show here that RXRα also has a broadly distributed expression, while RXRγ is confined to Area X – a region completely devoid of zRalDH transcript. Our findings are consistent with the notion that the zRalDH enzyme is transported from HVC and/or lMAN to Area X and RA, where locally generated ATRA could then act at the receptors present in these target regions.

**Figure 16:**
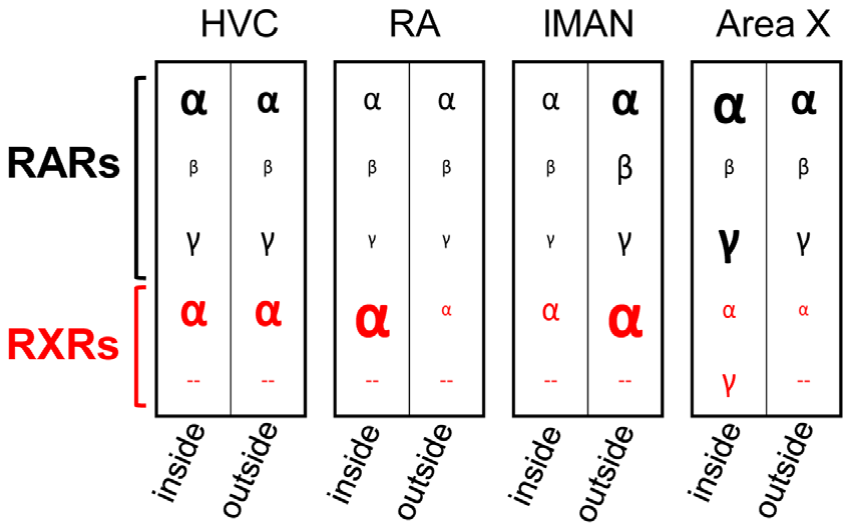
Overlapping expression of RARs and RXRs differ across song control nuclei, as well as between most song nuclei and their surroundings. Expression strength of all receptors based on visual estimates are schematically represented by symbol size (for RARs, data are from Jeong et al. (2005)). For all song nuclei except HVC, the receptor expression profile differs from the surroundings. Thus, each song nucleus has a unique combination of RAR(s)/RXR(s), which may lead to nucleus-specific sets of ATRA effects.

Expression of at least one RXR and one RAR overlapped in each one of the telencephalic song nuclei, qualifying them as potential targets of retinoid controlled gene transcription. The two groups of retinoid-related receptors, RXRs and RARs, are thought to mediate ATRA induced target gene transcription upon heterodimerization only [30]–[34]. But note that RXRs also hetero-dimerize with a multitude of other nuclear receptors, transcription factors and other peptides (reviewed by Lefebvre [51]). Thus, RXR mRNA distribution reflects a high functional diversity, and RXRs present in any region may serve other functions than ATRA-mediated signaling, irrespective of local ATRA presence. We did not investigate cellular colocalization of RXRs, RARs, and zRalDH. Except for RXRγ, however, receptors were expressed in most cells within the respective song nuclei, so that their expression is likely to overlap. We observed that: (1) All song nuclei but HVC exhibit RAR/RXR receptor expression profiles that differ from the surrounding tissue, and. (2) Each song nucleus has its own receptor expression profile (fig. 16). The three pallial song nuclei HVC, lMAN, and RA predominantly express RARα and RXRα, with lower levels in lMAN. HVC expresses RARγ [29]. RA has stronger RXRα expression than HVC and lMAN. The striatal Area X differs from the pallial nuclei most notably in that it expresses very little RXRα, but it also expresses RXRγ in a sparsely distributed population of large cells (fig. 6.B,C). They might correspond to the tonically-active fast-spiking pallidal cells in Area X, which provide input to the avian versions of the direct and indirect mammalian basal ganglia pathways [52], [53].

In lMAN, RXRα expression levels show considerable variation, which our data linked to time of day and age. However, additional factors which we did not control for may also influence RXRα levels in lMAN. One possibility is the social environment: some of the birds were moved across different groups of males, or housed at different times in smaller groups or large aviaries prior to RXRα expression assessments. Exposure to different songs of cage mates can lead, even in adult zebra finches with crystallized songs, to syllable modifications [54]. Such changes require lMAN [55], [56], and might be linked to RXRα-mediated processes in that nucleus.

An important finding is the difference between zRalDH transcript and protein within HVC: We detected zRalDH enzyme in HVC_RA_ projection neurons, yet these neurons do not contain zRalDH transcript [21]. zRalDH mRNA within HVC has only been reported for Area X-projecting neurons, which raises the question of how the zRalDH enzyme is present in RA-projecting cells. A similar dissociation of the cellular distributions of mRNA and peptide in HVC has been previously described for IGF-II [57].

While zRalDH *transcript* distribution was considerably more confined than ATRA distribution, zRalDH *protein* distribution did match ATRA distribution overall (fig. 9 and fig. 10). Only few regions were ATRA-positive and zRalDH peptide-negative, e.g. the anterior mesopallium close to lMAN, and parts of the striatum around Area X. These sites of ATRA presence might possibly be accounted for by diffusion, or by activity of ATRA synthesizing enzymes other than zRalDH, for instance CYP1B1. We conclude that within the song control system, ATRA distribution is accounted for by zRalDH enzyme transport over long distances to specific targets, but in other parts of the brain, ATRA diffusion or ATRA synthesis by enzymes other than zRalDH may also occur.

Taken together, these findings reveal unexpected complexities of ATRA related signaling, where there may be several ATRA targets across the different song nuclei, and suggest that ATRA actions may differ among the different song nuclei, leading potentially to differential target gene regulation in different nuclei. The downstream cellular effects can be autocrine or paracrine in nature, as suggested by RAR and zRalDH expression data [29].

### zRalDH enzyme trafficking in the song system

What is the likely source of zRalDH protein in RA and Area X? zRalDH mRNA is expressed in both HVC and lMAN. In the present study we found that retrogradely labeled neurons in both HVC and in lMAN from tracer injections in RA are also immunopositive for zRalDH. Within HVC, however, HVC_RA_ projection neurons do not express zRalDH transcript (only HVC_AreaX_ neurons do [21]), thus a transfer of either zRalDH transcript or protein across cell types would be required; cell type specificity of zRalDH mRNA expression has not yet been assessed in lMAN. To date, RalDH transcript transfer between cells has not been described, but a case of RalDH2 protein transfer between cells has been described (RalDH2 is homologous to zebra finch zRalDH): rat tanycytes do not express RalDH2 mRNA but contain RalDH2 protein, most likely taken up from the cerebro-spinal fluid [58]. Alternatively, many HVC neurons are organized in tight clusters, and dye coupling across cells has been observed for both HVC_RA_ and, more rarely, HVC_AreaX_ neurons [59]. HVC has gap junctions [60] that have been hypothesized to mediate dye transfer, but peptides or nucleic acids would be too large for them, thus the possible transfer mechanism remains undefined.

The strong immunohistochemical labeling of fiber tracts between HVC and RA and the fact that HVC lesions almost completely deplete RA of ATRA suggest that ATRA in RA depends on zRalDH protein that originates in HVC. An alternative source is lMAN, as lMAN_RA_ axon fibers traverse close to HVC, so that some of the zRalDH positive fiber in the tracts between HVC and RA could belong to lMAN_RA_ neurons. Consequently, both surgeries used for lesioning HVC may have affected axon bundles from lMAN to RA [61], [62]. Furthermore, in the reporter assay, ATRA-induced LacZ was detectable along the fiber tracts originating in lMAN and extending caudally. These tracts were unlikely to be confounded with HVC_RA_ projection neurons. Finally, we also found considerable amounts of ATRA-induced LacZ in female RA (data not shown). HVC is an unlikely source of this ATRA, since female HVC is small and not connected to RA. Thus, while not conclusive, the female data suggest that lMAN_RA_ projection neurons can be a source of ATRA in RA.

ATRA in Area X could also originate in HVC and/or lMAN, but here the evidence is more indirect. HVC_AreaX_ projection neurons are positive for zRalDH transcript [21] and could provide Area X with the enzyme. Further, we show here that lMAN cells that project to RA contain zRalDH enzyme. Since lMAN contains cells that project to both RA and Area X [63], this opens up the possibility that lMAN projection neurons also provide Area X with zRalDH protein.

### A possible role for ATRA in RA

The ATRA distribution in the song system suggested by our experiments is consistent with a possible role for balancing two neural pathways that are essential for song, the vocal motor pathway comprising HVC and RA on the one hand, and the AFP with Area X and lMAN on the other hand (fig. 1). A number of studies suggest that the way a bird introduces lasting changes to his song is through balancing these two pathways: Activation of the posterior motor pathway produces stereotyped firing (and song) patterns, while activation of the AFP results in increased variability of vocal output [64]–[67]. The bird uses AFP-generated random exploration to eventually develop a stereotyped song that is matching an internal sensory template [65], [67]–[71]. Song nucleus RA is an important interface between the “stereotyped” posterior and the “exploratory” anterior pathways, receiving the stereotyped firing patterns from the HVC_RA_ projections, while the lMAN_RA_ projections contribute the variable input (see fig. 1). Successful imitation of the tutor's song patterns by the juvenile bird is dependent on the exploratory AFP-driven pattern production: early lMAN lesions lead to a simple, stereotyped, prematurely crystallized song [72]. lMAN-dependent exploration is still available to adult birds to some extent, and can be used for dynamic re-adjustment of the motor output even after song crystallization [55], [73], [74]. Both lMAN and HVC could, by controlling the amount of ATRA they provide to nucleus RA, locally modify synapses within RA, and thereby regulate the respective contributions of the AFP and the motor pathway to RA activity. Indeed, lMAN projections onto RA have been shown to influence synaptic connectivity of the descending HVC_RA_ inputs in juvenile birds: lMAN lesions lead to a sudden numeric *decrease* of HVC_RA_ synapses, along with a *strengthening* of their excitatory transmission [75]. Synaptic terminals of the HVC_RA_ neurons contain protein kinase C (PKC) [76], [77], a molecule involved in synaptic plasticity and a direct target for ATRA [78], [79], and thus a candidate mechanism for ATRA-controlled restructuring of synapses in RA. However, other ways of ATRA controlled synaptic alterations have been described and might be at play here as well, such as regulating dendritic mRNA translation to induce rapid dendritic growth, as in mouse hippocampal neurons [80]–[83], or rapid AMPA or GABA_A_ receptor trafficking at the synapse [6], [82], [84]–[86]. This latter process is mediated by a nontranscriptional function of the RARα receptor, which is expressed in RA [29]. This mode of action of ATRA is thus an interesting candidate mechanism for balancing stereotyped inputs from HVC and more variable inputs from lMAN, by controlling synapse strength in RA.

Interestingly, feeding juvenile zebra finches supplemental ATRA increases the variability of the song when the birds reach adulthood, and at the same time leads to a correlated increase of gene expression levels in lMAN and Area X [87] – a finding that is consistent with a possible role of ATRA in balancing the stereotyped vs. exploratory pathways. As birds age, we found that zRalDH enzyme in lMAN decreases, which is consistent with the notion that as ATRA levels decline at the lMAN-RA synapse, less variability is reaching RA and the influence of HVC becomes stronger. This could in turn lead to more stereotyped song, which is what happens as birds get older, even in adults [88], [89].

Our observations that RXRα transcript in lMAN undergoes circadian changes, and in addition decreases with age, are compatible with this scenario. RXRα transcript in lMAN is highest at young age, and in addition, it changes in a circadian manner, increasing after waking up and decreasing before the end of the day. While these fluctuations happen over very different time scales – one over the course of a day and the other over the course of several weeks or months – they are paralleled by the rate of song change: The strongest changes to a birds' song towards stereotypy happen at early age, when RXRα in lMAN is generally high. The strongest changes in syllable structure are observed in the first 2–3 hours of singing after waking up, the syllables being less structured in the morning as compared to the previous evening, but regaining structure throughout the hours of morning singing [90]. This observation has been made in juvenile birds, but while the oscillations in syllable structure decrease with age, they might still persist to some extent in the adult bird. The time resolution of our circadian analysis is not fine enough to decide whether RXRα transcription and the rate of song structure-change peak at exactly the same time, or one is following the other. However, the fluctuations of RXRα transcription in lMAN happen in a similar rhythm or time scale as the rate of song modification. This would be in line with a possible role of RXRα-mediated ATRA signaling in “translating” those of the exploratory neural/song patterns that correspond to the desired template song into long lasting synaptic alterations. Increasing RXRα after waking up in the morning could help to consolidate synaptic states that correspond to the desired syllable structure both within lMAN and at the lMAN-to-RA synapse. A decrease in RXRα expression with age would be in line with the decreasing need for exploration-driven song changes (and thus for consolidating the desired exploratory patterns) as the song matures.

This scenario for RXRα function in lMAN is speculative so far, and needs further experimental research to be verified.

### ATRA signaling in higher auditory areas

The CYP26B1 and zRalDH expression patterns revealed the higher auditory regions CMM/CLM and NCM as possible targets of complex ATRA signaling. Additionally, CYP26B1 is, to our knowledge, the first molecular marker for CM. The lack of overlap between CYP26B1 and zRalDH expression (fig. 14 and fig. 15) suggests that the role of CYP26B1 in the zebra finch brain consists in establishing regional sinks or gradients of ATRA.

NCM and CMM show high immediate early gene expression after the awake, behaving bird has heard song, and these regions are likely to be involved in song memorization required for song learning and perceptual discrimination [91]. The differential, partly gradient-like expression patterns of CYP26B1 in CM and of zRalDH in and adjacent to NCM suggest differential ATRA presence across these areas. The results of our reporter assay are consistent with ATRA increasing along the dorso-ventral axis in CM, and decreasing along the antero-posterior axis in NCM, perhaps in a gradient-like way. Determining the exact nature of this differential distribution would require a quantitative ATRA detection method, such as developed by Shimozono et al. [92] with genetically encoded fluorescent ATRA indicators.

Note that the origin of the mesopallial ATRA is unclear, since neither zRalDH transcript nor enzyme are present there. ATRA might diffuse from either hyper- or nidopallium to its mesopallial sites, or be synthesized by an enzyme other than zRalDH. Receptor expression patterns suggest in addition that the higher auditory regions may respond in a complex way to this ATRA distribution (e.g. RARβ-mediated responses being stronger in the anterior part of CMM than in the rest of the higher auditory areas). To our knowledge, no similar gradients of zRalDH, or retinoic acid degrading enzymes have been described to date for adult mammalian brains, although they are well documented in embryonic development [92]–[95]. There, ATRA gradients contribute to embryogenesis and differentiation by creating regional differences in the expression of retinoid target genes, which then contribute to local tissue differentiation and patterning [1]. In the adult brain, expression gradients of the enzymes that generate and degrade ATRA might perhaps play a similar role for regional differentiation and function: They might result in differential local ATRA presence, or perhaps gradients, as suggested by the observed ATRA-induced reporter distribution in the cell coculture assay. In turn, they could contribute to regional differences in neuronal phenotypes and function (e.g. in the different parts of the auditory system).

Expression gradients in the postnatal brain have been described for a limited number of other genes, e.g. in the rodent hippocampus, where collagen gene Col15a1, crystalline (Crym), integrin subunit α8, and kainate receptor subunits GluR-6 and KA-1 are expressed in a gradient-like manner in fields C1 and C3 [96]–[100], but the function of these postnatal expression gradients is unknown.

In the developing brain, opposing gradients of the EphA receptor and ephrinA, its ligand, are involved in topographic mapping, for instance of the retinotectal projection (reviewed by Klein and Kania [101]). The reciprocal distributions of ATRA-induced LacZ and CYP26B1 in CM are reminiscent of these opposing gradients. Whether or not they may be involved in CM topography is up to further investigation.

Interestingly, retinoid signaling might play a role in the early postnatal development of the mouse auditory cortex, as mouse RalDH3 is transiently upregulated in this region during an early postnatal phase [102], [103]. Whether or not mouse CYP26B1 is also regulated at this site and time window is not known. Contrasting to the songbird findings, however, neither mouse RalDH nor CYP26B1 or any of the other retinoic acid degrading CYP26s seem to be expressed in the *adult* mouse auditory cortex (see Wagner et al. (2002), and Allen Brain Atlas; http://mouse.brain-map.org). CYP26B1 expression in the adult mouse brain is limited to the amygdala, parts of the hippocampal formation, and the adjacent subiculum (Allen Brain Atlas).

Prolonged zRalDH expression in zebra finches' higher auditory areas might point to prolonged auditory plasticity in birds. Expression of immediate early genes such as ZENK induced by auditory song exposure in adult zebra finches' higher auditory areas is long known [44], [104]–[106], and an electrophysiological study has shown that adult neuronal plasticity takes place within these areas [107]: “Tuning” of NCM neurons – i.e. the sound frequencies they respond best to – is shown here to be influenced by recent auditory experience. This example of a non-developmental locally differential ATRA presence may be an interesting target for future research.

With this study, we provide further evidence for a prominent role of retinoid signaling in the brain of an adult vertebrate. Our results suggest that retinoid signaling in the songbird brain shows a previously unknown spatial complexity, with ATRA likely acting at sites that are distant from areas that express the synthetic enzyme. Our findings complement previous findings to generate a comprehensive picture of likely targets of action for ATRA in the songbird brain and song system, inviting future mechanistic studies investigating the long range function of retinoid signaling and its possible role for a post-natally acquired vocal motor behavior.

## Supporting Information

Figure S1 **Zebra finch RXRα and RXRγ domain structure and position of probes used for** ***in situ*** **hybridization (ISH).** Light gray bars represent the ORF, darker segments functional domains, arrows exons. RXRs are characterized by a zinc finger towards the 5′ end of the gene, and a hormone receptor domain towards its 3′ end. Start and end nucleotides of the ORF and domains are indicated by numbers. The zinc finger domains of the two genes are aligned vertically. Black lines underneath represent the probes used for ISH. A: For RXRα, a 658 bp probe overlapping the 3′ UTR was used. B: For RXRγ, identity to RXRα is indicated in percent for each part of the gene. Two different transcriptional variants are known for RXRγ in chicken. The ORFs of these different variants are symbolized by narrow, light bars beneath the RXRγ bar. The only zebra finch RXRγ we found corresponds to the shorter variant's sequence. We cannot exclude that the other transcriptional variant also exists in zebra finches. Our probe would not distinguish between the two variants. The RXRγ 738 probe yielded a distinct expression pattern different from RXRα.
(TIF)

Figure S2 **Positioning of ISH probes for the ATRA degrading cytochrome genes.** Pink bars represent coding sequences (CDS), and in case of CYP26A1, an additional antisense open reading frame [ORF]; regions coding for p450 domains which are specific for this class of cytochromes are marked in dark red. Regions covered by probes are represented as green arrows. A: For CYP26A1, we used two different probes, one covering a region close to the 5′ end of the CDS which falls into a potential additional antisense ORF, and one covering a region further downstream. B: Our CYP26B1 probe covered a region close to the 5′ end of the CDS. C: As the zebra finch CYP26C1 sequence is unknown, a putative CYP26C1 sequence predicted by automated computational analysis of the zebra finch genome is shown (NCBI Reference Sequence: XM_002189751.1). We used a probe near the middle of this predicted gene.
(TIF)

Figure S3 **An antibody against human ALDH1A2, the human homolog to zRalDH, specifically labels zebra finch zRalDH.** A: Parasagittal zebra finch brain section immunolabeled with αALDH1A2 antibody visualized with DAB staining. B (control 1): Without primary antibody, no staining occurs. C and D (control 2): No staining occurs after preincubation of sections with ALDH1A2 blocking peptide (C: brightfield view, D: darkfield view; arrows indicate song nuclei HVC and RA). In all photos, frontal is to the right and dorsal is up. Scale bar  = 1 mm.
(TIF)

Figure S4 **Thalamic and midbrain expression of zebra finch RXRs.** A, C, E: Drawings of the thalamic part of frontal brain sections shown in B, D, F. B, D, F: RXRα expression in the thalamus by ISH, from frontal to caudal. Nucleus spiriformis medialis and lateralis (SpL, SpM) showed the strongest RXRα labeling. Labeled cells were also found in the ventral tegmental area (VTA), substantia nigra (SN), and the optic tectum. G: Comparison of thalamic RXRα and RXRγ expression (parasagittal sections, frontal is to the right). Drawing on the left indicates regions shown in the right photos. RXRα expression is shown in middle, RXRγ expression on the right. Both RXRs are highly expressed in nucleus spiriformis medialis, whereas the remaining thalamus shows little (RXRα) or no (RXRγ) labeling. For abbreviations, see table 1. Scale bars  = 0.5 mm.
(TIF)

Figure S5 **Retrospective review of zRalDH immunostainings and ISHs suggests that zRalDH expression in lMAN decreases with age.** Dashed rectangle in drawing on top indicates approximate region shown in A and B. A: Immunolabeling of zRalDH protein in lMAN and surrounding of four animals of different ages. Dashed circle surrounds lMAN. Density of immunolabeling in lMAN decreases as age increases, although some cells are still strongly labeled at high age. B: zRalDH ISH showing expression around lMAN region; dashed circle surrounds lMAN. Like zRalDH protein, zRalDH mRNA in lMAN is decreased in an aged animal as compared to a juvenile, due to lower density of labeled cells. In all panels, frontal is to the right and dorsal is up. Scale bar  = 1 mm.
(TIF)

Figure S6 **CYP26B1 is expressed in neuronal population(s) with medium to high density.** A: Drawing indicates the CMM region shown in photos B–E. B and C: CYP26B1 expression by ISH (B), counterstained with DAPI to visualize cell nuclei (C). B shows the dorsoventral CYP26B1 expression gradient in CMM, a comparison to cell density in C shows that the density of the CYP26B1 positive cell population is medium high. D and E: Bright field and fluorescence views of CYP26B1 ISH immunostained for the neuronal marker Hu (red), and counterstained with DAPI (blue). CYP26B1 positive cells are also Hu positive (white arrowheads). F–H: CYP26B1 expression in the medial habenula. F shows CYP26B1 by ISH, G is the according DAPI stain, and F the merged image. Most cells particularly at the margins of the medial habenula express CYP26B1. Scale bars for B and C = 50µm, for D and E = 20µm, for F–H = 100µm.
(TIF)

Figure S7 **In the mesopallium, ATRA-induced reporter decreases along the antero-posterior axis.** This is consistent with CYP26B1 expression but requires either ATRA diffusion from hyper- or nidopallium, or mesopallial ATRA synthesis by some other ATRA synthesizing enzyme such as CYP1B1. Schematic drawing on top left depicts the approximate area shown in panels A and B. A: zRalDH and CYP26B1 expression by *in situ* hybridization. Gray dashed boxes outline region shown in panel B. Note that zRalDH and CYP26B1 expression are non-overlapping. B: ATRA distribution as determined by ATRA reporter cell culture assay. Blue label indicates ATRA-induced gene expression. The upper picture is a summing-up overview of ATRA distribution in the dorso-caudal area (gray indicates ATRA activity), below are corresponding examples of 5 different animals. The dorso-caudal mesopallium is devoid of ATRA-induced reporter, consistent with CYP26B1 expression and lack of zRalDH expression (red arrowheads). As CYP26B1 expression decreases towards the more rostro-ventral mesopallium, ATRA-induced reporter increases. For more pictures of CM expression of CYP26B1 and zRalDH in different mediolateral planes, see fig. 15.
(TIF)

Table S1 **Number and age of animals used for ISH experiments, and treatments they underwent.**
(DOCX)

Table S2 **Number and age of animals used in reporter cell assay experiments, and treatments they underwent.**
(DOCX)

Table S3 **Number and age of animals used for zRalDH immunohistochemistry experiments, and treatments they underwent.**
(DOCX)
