# Supplementary figures and images for: Long-Distance Retinoid Signaling in the Zebra Finch Brain
Source: PLoS One. 2014 Nov 13;9(11):e111722. doi: 10.1371/journal.pone.0111722 (PMC4230966; doi:10.1371/journal.pone.0111722)

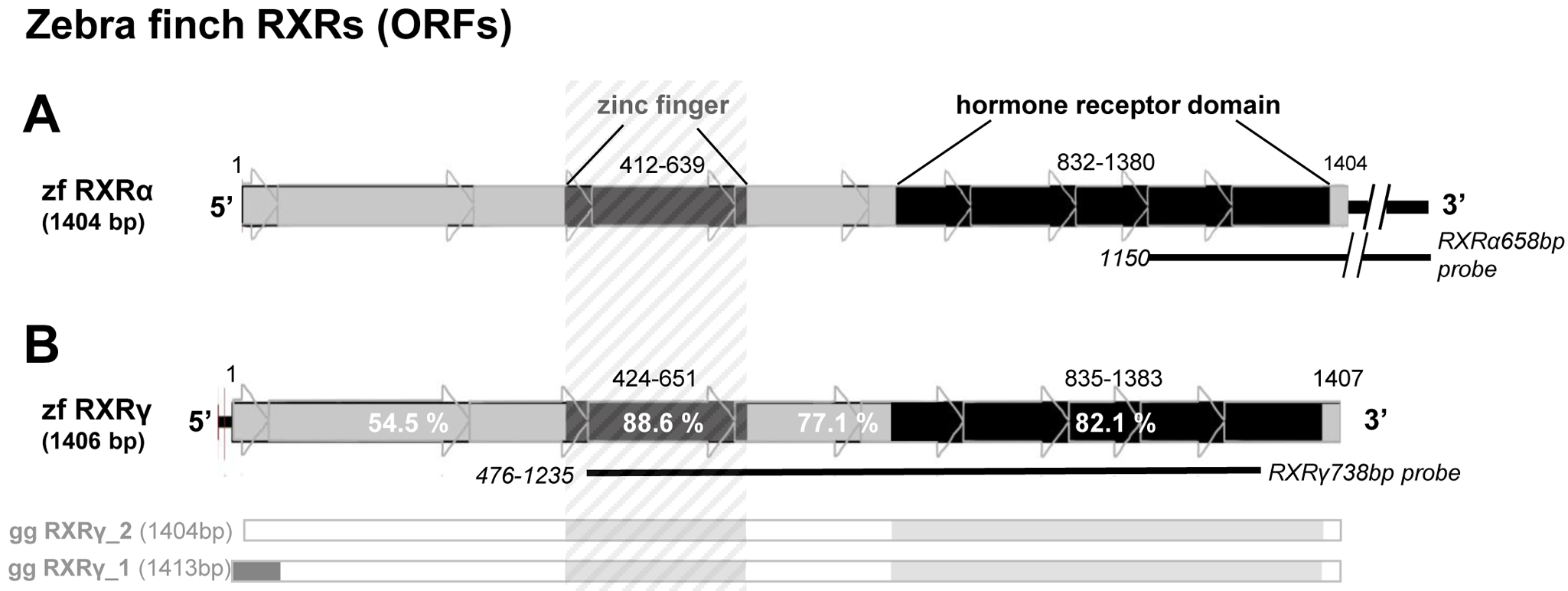

Supplement: Figure S1 — Zebra finch RXRα and RXRγ domain structure and position of probes used for in situ hybridization (ISH). Light gray bars represent the ORF, darker segments functional domains, arrows exons. RXRs are characterized by a zinc finger towards the 5′ end of the gene, and a hormone receptor domain towards its 3′ end. Start and end nucleotides of the ORF and domains are indicated by numbers. The zinc finger domains of the two genes are aligned vertically. Black lines underneath represent the probes used for ISH. A: For RXRα, a 658 bp probe overlapping the 3′ UTR was used. B: For RXRγ, identity to RXRα is indicated in percent for each part of the gene. Two different transcriptional variants are known for RXRγ in chicken. The ORFs of these different variants are symbolized by narrow, light bars beneath the RXRγ bar. The only zebra finch RXRγ we found corresponds to the shorter variant's sequence. We cannot exclude that the other transcriptional variant also exists in zebra finches. Our probe would not distinguish between the two variants. The RXRγ 738 probe yielded a distinct expression pattern different from RXRα. (TIF) [file pone.0111722.s001.tif]

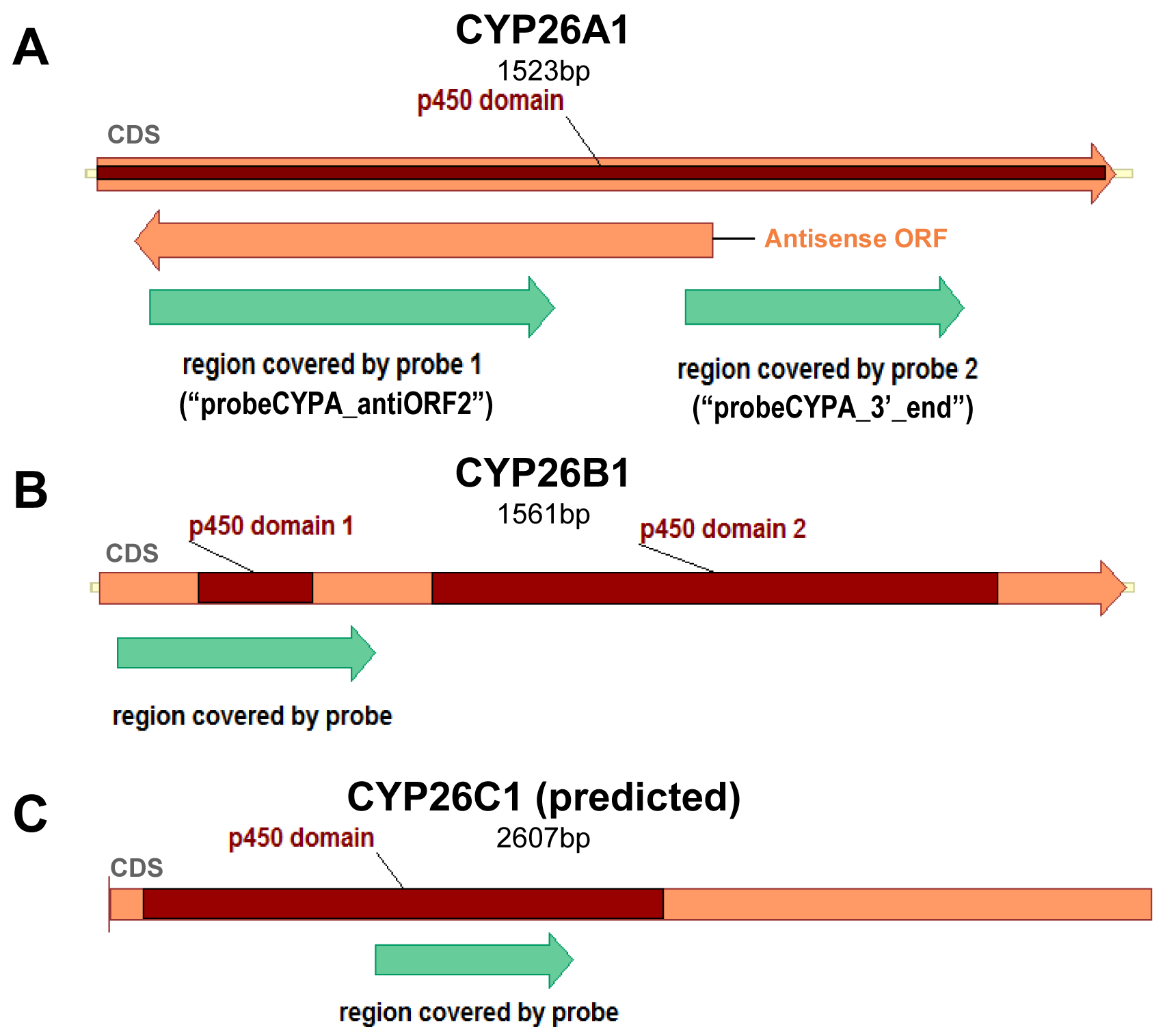

Supplement: Figure S2 — Positioning of ISH probes for the ATRA degrading cytochrome genes. Pink bars represent coding sequences (CDS), and in case of CYP26A1, an additional antisense open reading frame [ORF]; regions coding for p450 domains which are specific for this class of cytochromes are marked in dark red. Regions covered by probes are represented as green arrows. A: For CYP26A1, we used two different probes, one covering a region close to the 5′ end of the CDS which falls into a potential additional antisense ORF, and one covering a region further downstream. B: Our CYP26B1 probe covered a region close to the 5′ end of the CDS. C: As the zebra finch CYP26C1 sequence is unknown, a putative CYP26C1 sequence predicted by automated computational analysis of the zebra finch genome is shown (NCBI Reference Sequence: XM_002189751.1). We used a probe near the middle of this predicted gene. (TIF) [file pone.0111722.s002.tif]

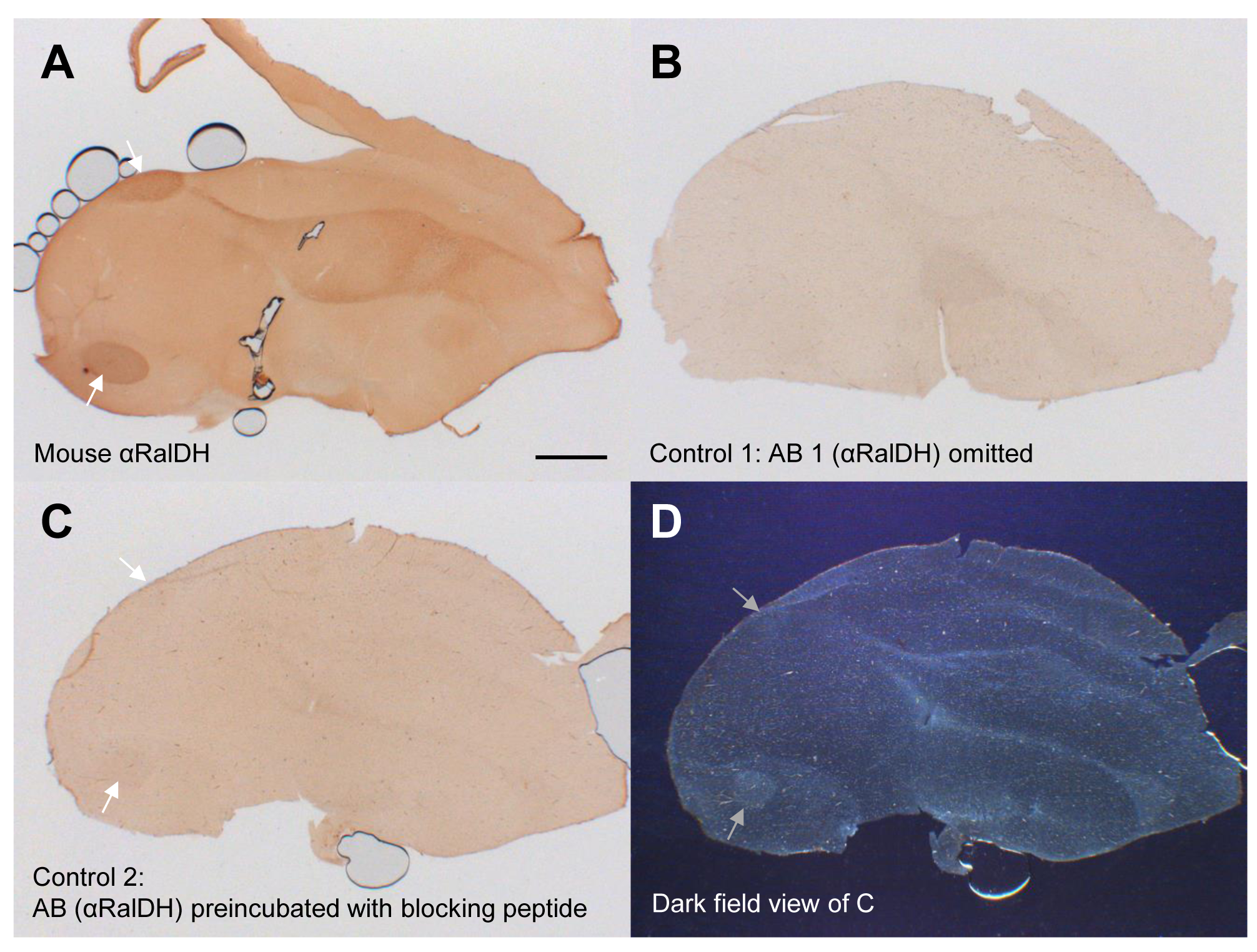

Supplement: Figure S3 — An antibody against human ALDH1A2, the human homolog to zRalDH, specifically labels zebra finch zRalDH. A: Parasagittal zebra finch brain section immunolabeled with αALDH1A2 antibody visualized with DAB staining. B (control 1): Without primary antibody, no staining occurs. C and D (control 2): No staining occurs after preincubation of sections with ALDH1A2 blocking peptide (C: brightfield view, D: darkfield view; arrows indicate song nuclei HVC and RA). In all photos, frontal is to the right and dorsal is up. Scale bar = 1 mm. (TIF) [file pone.0111722.s003.tif]

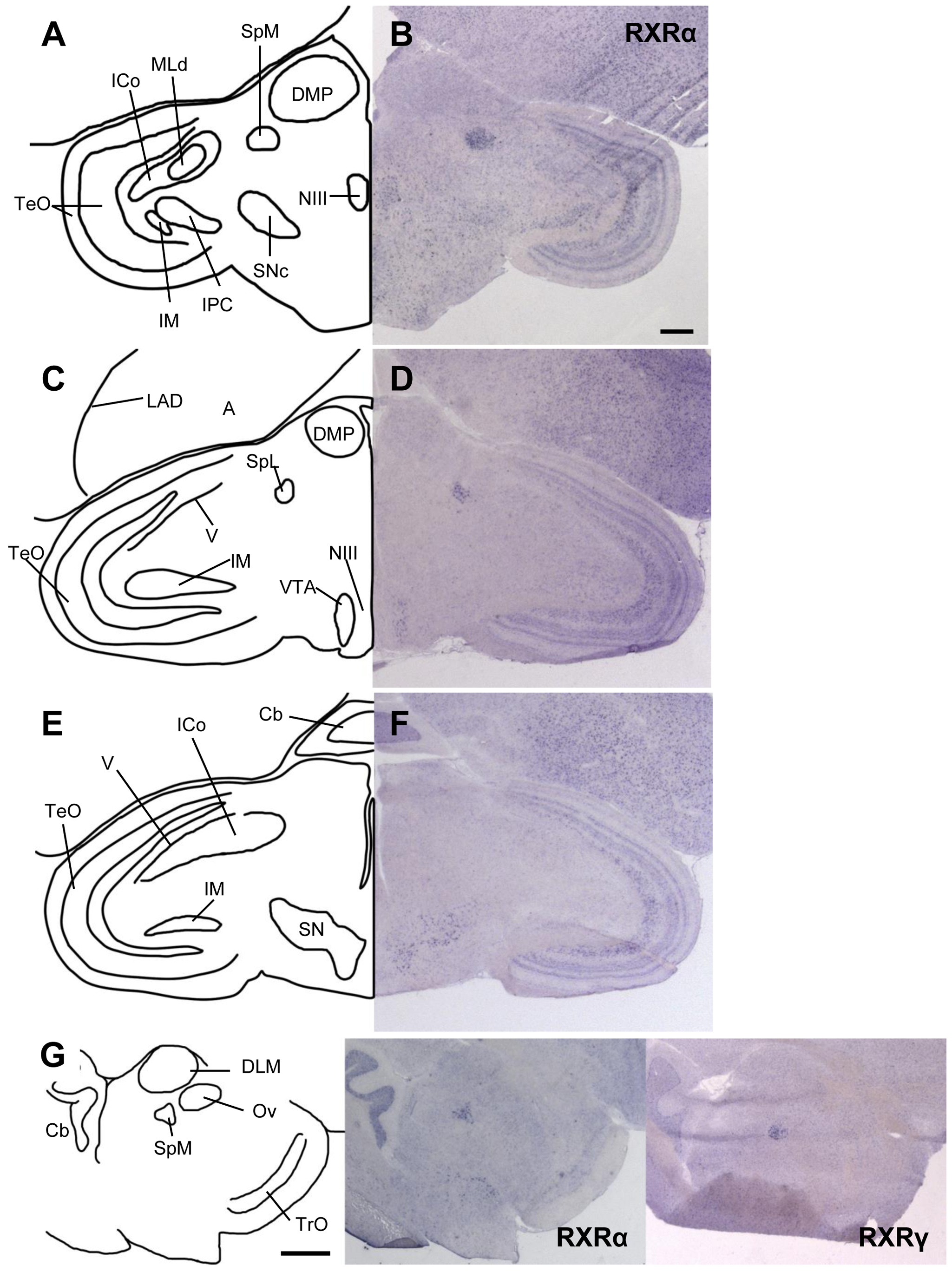

Supplement: Figure S4 — Thalamic and midbrain expression of zebra finch RXRs. A, C, E: Drawings of the thalamic part of frontal brain sections shown in B, D, F. B, D, F: RXRα expression in the thalamus by ISH, from frontal to caudal. Nucleus spiriformis medialis and lateralis (SpL, SpM) showed the strongest RXRα labeling. Labeled cells were also found in the ventral tegmental area (VTA), substantia nigra (SN), and the optic tectum. G: Comparison of thalamic RXRα and RXRγ expression (parasagittal sections, frontal is to the right). Drawing on the left indicates regions shown in the right photos. RXRα expression is shown in middle, RXRγ expression on the right. Both RXRs are highly expressed in nucleus spiriformis medialis, whereas the remaining thalamus shows little (RXRα) or no (RXRγ) labeling. For abbreviations, see table 1. Scale bars = 0.5 mm. (TIF) [file pone.0111722.s004.tif]

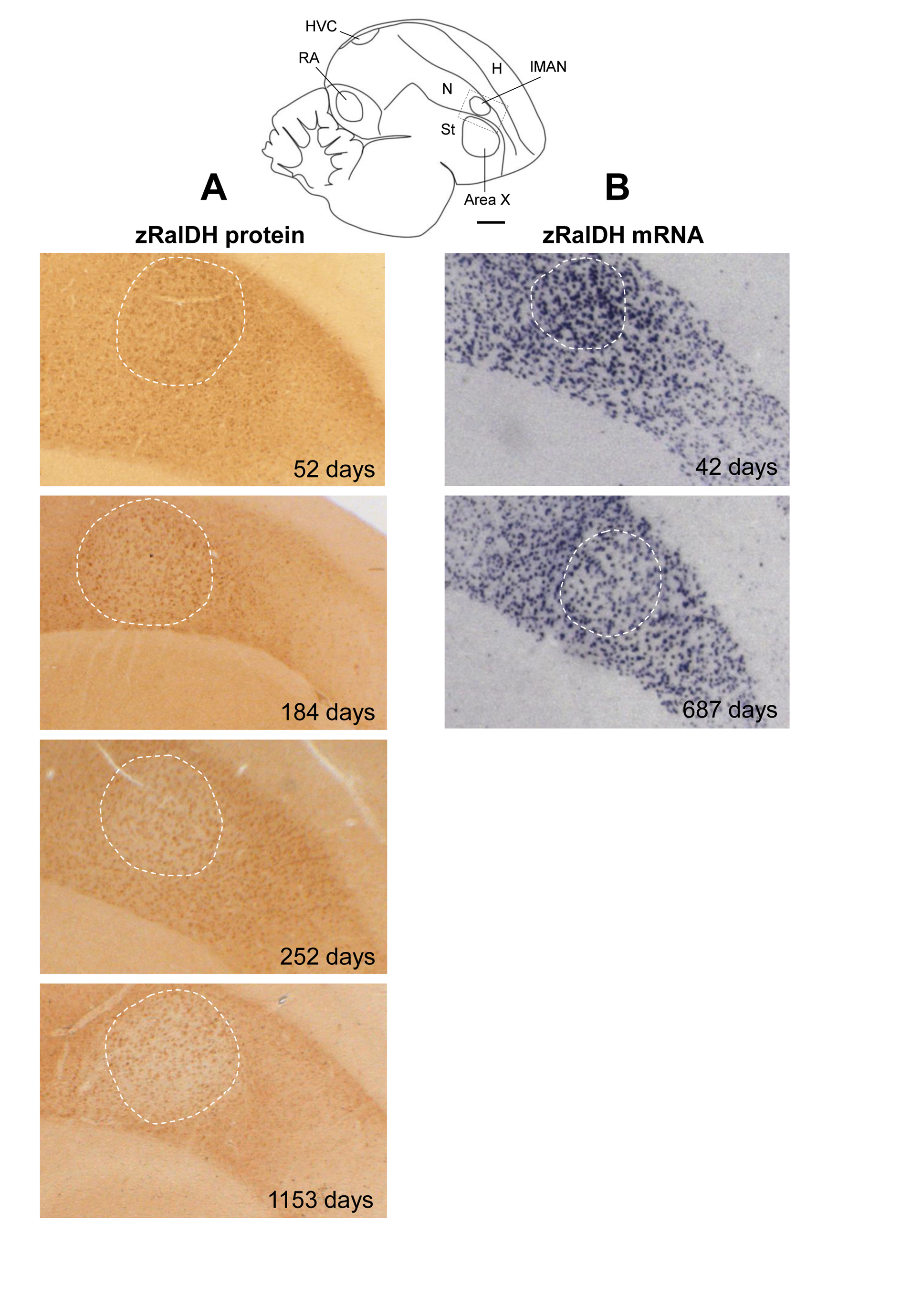

Supplement: Figure S5 — Retrospective review of zRalDH immunostainings and ISHs suggests that zRalDH expression in lMAN decreases with age. Dashed rectangle in drawing on top indicates approximate region shown in A and B. A: Immunolabeling of zRalDH protein in lMAN and surrounding of four animals of different ages. Dashed circle surrounds lMAN. Density of immunolabeling in lMAN decreases as age increases, although some cells are still strongly labeled at high age. B: zRalDH ISH showing expression around lMAN region; dashed circle surrounds lMAN. Like zRalDH protein, zRalDH mRNA in lMAN is decreased in an aged animal as compared to a juvenile, due to lower density of labeled cells. In all panels, frontal is to the right and dorsal is up. Scale bar = 1 mm. (TIF) [file pone.0111722.s005.tif]

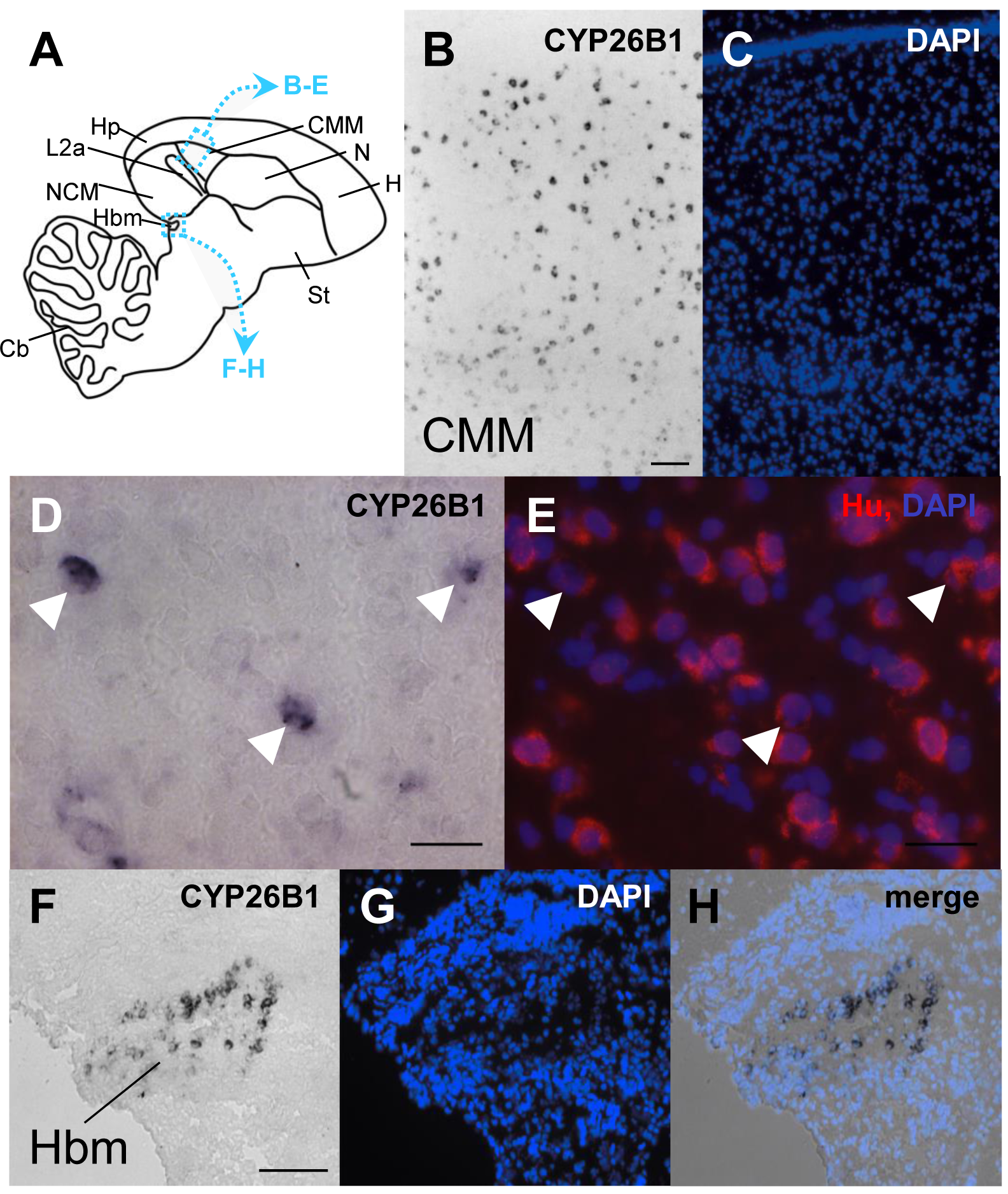

Supplement: Figure S6 — CYP26B1 is expressed in neuronal population(s) with medium to high density. A: Drawing indicates the CMM region shown in photos B–E. B and C: CYP26B1 expression by ISH (B), counterstained with DAPI to visualize cell nuclei (C). B shows the dorsoventral CYP26B1 expression gradient in CMM, a comparison to cell density in C shows that the density of the CYP26B1 positive cell population is medium high. D and E: Bright field and fluorescence views of CYP26B1 ISH immunostained for the neuronal marker Hu (red), and counterstained with DAPI (blue). CYP26B1 positive cells are also Hu positive (white arrowheads). F–H: CYP26B1 expression in the medial habenula. F shows CYP26B1 by ISH, G is the according DAPI stain, and F the merged image. Most cells particularly at the margins of the medial habenula express CYP26B1. Scale bars for B and C = 50µm, for D and E = 20µm, for F–H = 100µm. (TIF) [file pone.0111722.s006.tif]

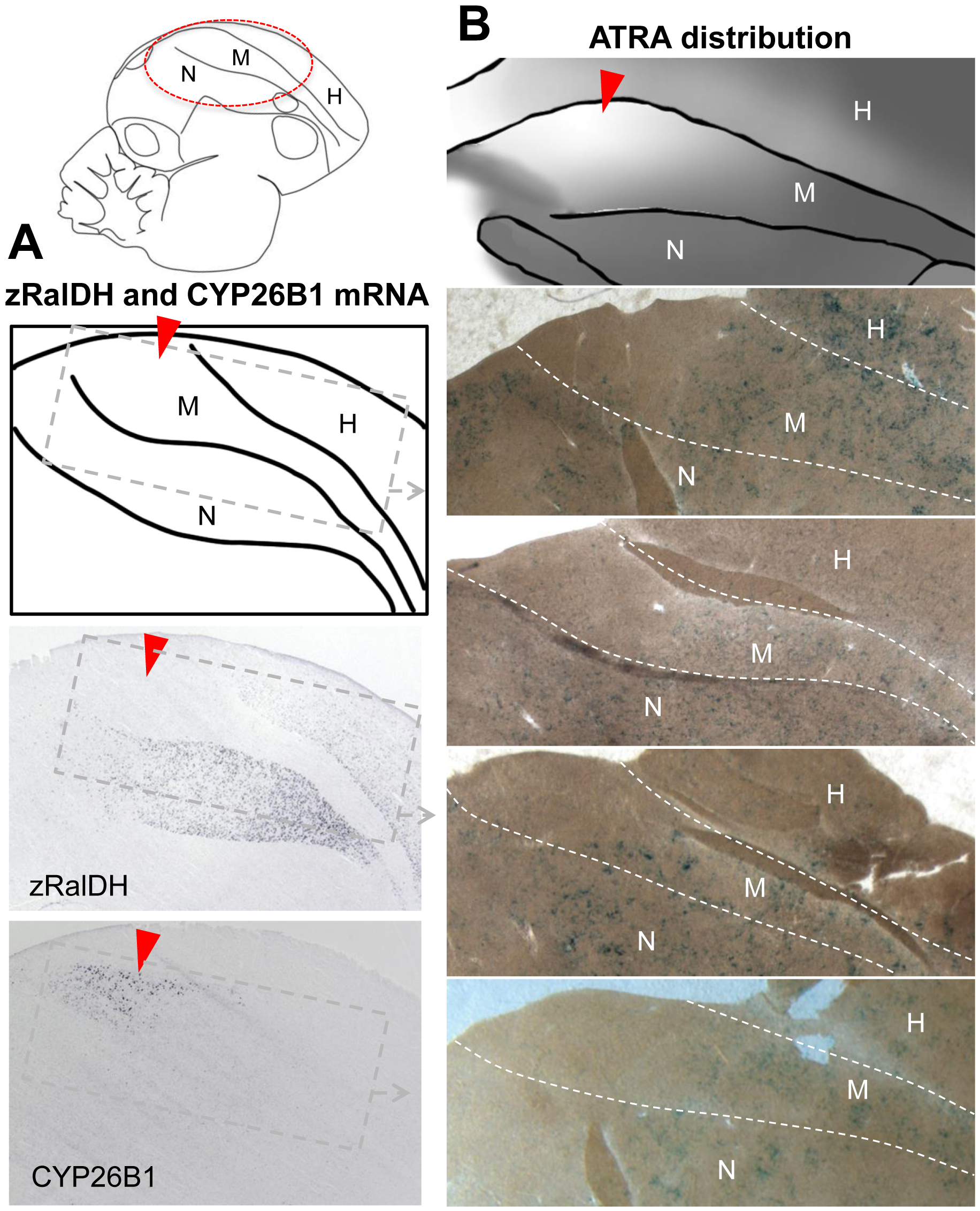

Supplement: Figure S7 — In the mesopallium, ATRA-induced reporter decreases along the antero-posterior axis. This is consistent with CYP26B1 expression but requires either ATRA diffusion from hyper- or nidopallium, or mesopallial ATRA synthesis by some other ATRA synthesizing enzyme such as CYP1B1. Schematic drawing on top left depicts the approximate area shown in panels A and B. A: zRalDH and CYP26B1 expression by in situ hybridization. Gray dashed boxes outline region shown in panel B. Note that zRalDH and CYP26B1 expression are non-overlapping. B: ATRA distribution as determined by ATRA reporter cell culture assay. Blue label indicates ATRA-induced gene expression. The upper picture is a summing-up overview of ATRA distribution in the dorso-caudal area (gray indicates ATRA activity), below are corresponding examples of 5 different animals. The dorso-caudal mesopallium is devoid of ATRA-induced reporter, consistent with CYP26B1 expression and lack of zRalDH expression (red arrowheads). As CYP26B1 expression decreases towards the more rostro-ventral mesopallium, ATRA-induced reporter increases. For more pictures of CM expression of CYP26B1 and zRalDH in different mediolateral planes, see fig. 15. (TIF) [file pone.0111722.s007.tif]
