# Supplementary material for: Long-Distance Retinoid Signaling in the Zebra Finch Brain
Source: PLoS One. 2014 Nov 13;9(11):e111722. doi: 10.1371/journal.pone.0111722 (PMC4230966; doi:10.1371/journal.pone.0111722)
Supplement: Table S1 — Number and age of animals used for ISH experiments, and treatments they underwent. (DOCX) [file pone.0111722.s008.docx]

**Table S1: Overview over experimental birds used for *in situ*** hybridization**.**

| **Animals used for *in situ*** hybridization | | | |
| --- | --- | --- | --- |
| **Sex** | **age** | **number** | **treatment / experiment** |
| male | juvenile, 20 days | 2 | RXRα, RXRγ, CYP26A1, CYP26B1, CYP26C1 *in situ* hybridization |
| male | juvenile, 41 days | 1 | RXRα, RXRγ, CYP26A1, CYP26B1, CYP26C1 *in situ* hybridization |
| male | juvenile, 49 days | 1 | RXRα, RXRγ, CYP26A1, CYP26B1, CYP26C1 *in situ* hybridization |
| male | juvenile, 64-68 days | 4 | RXRα, RXRγ, CYP26A1, CYP26B1, CYP26C1 *in situ* hybridization |
| male | adult >150 days | 19 | RXRα, RXRγ *in situ* hybridization only |
| male | adult >150 days | 7 | RXRα, RXRγ, CYP26A1, CYP26B1, CYP26C1 *in situ* hybridization |
| female | adult >150 days | 2 | CYP26A1, CYP26B1, CYP26C1 *in situ* hybridization |
| unknown | embryo, equivalent to stage 20 chicken embryo (atlas by Hamburger & Hamilton, 1992) | 1 | RXRα, RXRγ, CYP26A1, CYP26B1, CYP26C1 *in situ* hybridization |
| total |  | 37 |  |
