# Supplementary material for: Long-Distance Retinoid Signaling in the Zebra Finch Brain
Source: PLoS One. 2014 Nov 13;9(11):e111722. doi: 10.1371/journal.pone.0111722 (PMC4230966; doi:10.1371/journal.pone.0111722)
Supplement: Table S2 — Number and age of animals used in reporter cell assay experiments, and treatments they underwent. (DOCX) [file pone.0111722.s009.docx]

**Table S2: Overview over experimental birds used in the ATRA reporter assay.**

| **Animals used in ATRA reporter assay** | | | |
| --- | --- | --- | --- |
| **Sex** | **age** | **number** | **treatment / experiment** |
| male | juvenile, 38-42 days | 1 | None |
| male | juvenile, 38-42 days | 2 | HVC lesioned unilaterally |
| male | adult > 240 days | 9 | None |
| male | adult > 240 days | 3 | HVC lesioned unilaterally |
| male | adult > 240 day | 2 | HVC_RA_ fibertract lesioned |
| female | adult > 240 day | 2 | None |
| total |  | 19 |  |
