# Supplementary material for: Long-Distance Retinoid Signaling in the Zebra Finch Brain
Source: PLoS One. 2014 Nov 13;9(11):e111722. doi: 10.1371/journal.pone.0111722 (PMC4230966; doi:10.1371/journal.pone.0111722)
Supplement: Table S3 — Number and age of animals used for zRalDH immunohistochemistry experiments, and treatments they underwent. (DOCX) [file pone.0111722.s010.docx]

**Table S3: Overview over birds used for zRalDH immunohistochemistry.**

| **Animals used in zRalDH immunohistochemistry experiments** | | | |
| --- | --- | --- | --- |
| **Sex** | **age** | **number** | **treatment / experiment** |
| male | juvenile 51-52 days | 2 | HVC lesioned unilaterally |
| male | adult 6-8.5 months | 4 | None |
| male | adult 7 months | 1 | HVC lesioned unilaterally |
| male | adult in 2^nd^ year | 3 | None |
| male | adult > 3 years | 2 | None |
| total |  | 12 |  |
